# Mitochondria–Endoplasmic Reticulum Crosstalk in Pancreatic β-Cell Stress: A Driver of Diabetes

**DOI:** 10.3390/cells15191733

**Published:** 2026-09-23

**Authors:** Sara Anjum Niinuma, Ashleigh Suzu-Nishio Takemoto, William Junior Khalil, Alexandra E Butler, Abu Saleh Md Moin

**Affiliations:** 1 School of Medicine, Royal College of Surgeons in Ireland-Medical University of Bahrain, Busaiteen 15503, Bahrain; 2 Research Department, Royal College of Surgeons in Ireland-Medical University of Bahrain, Busaiteen 15503, Bahrain

**Keywords:** mitochondrial-associated membranes, MAMs, β-cell stress, β-cell dysfunction

## Abstract

Pancreatic β-cell failure is central to the pathogenesis of type 2 diabetes (T2D), developing when chronic metabolic stress overwhelms the adaptive capacity of these cells. Because insulin biosynthesis and glucose-stimulated secretion depend on tightly coordinated organelle function, crosstalk between the endoplasmic reticulum (ER) and mitochondria strongly influences β-cell fate. The two organelles are physically coupled at mitochondria-associated membranes (MAMs), contact sites that synchronize ER Ca^2+^ release with mitochondrial Ca^2+^ uptake and adenosine triphosphate (ATP) production, and that help regulate redox balance, autophagy, mitophagy and the unfolded protein response. This review draws together evidence on three questions. First, which stresses converge on the β-cell: ER stress, oxidative stress and dysregulated autophagy and mitophagy. Second, how ER–mitochondria crosstalk sustains β-cell health through Ca^2+^ handling and the proteins that maintain MAM integrity, including inositol 1,4,5-trisphosphate (IP_3_) receptors, voltage-dependent anion channel 1 (VDAC1), mitofusin 2 (Mfn2) and the ER stress sensors IRE1α and PERK. Third, how glucolipotoxic and inflammatory stress remodels these contacts and pushes communication towards a maladaptive state marked by mitochondrial Ca^2+^ overload, ROS accumulation, defective organelle quality control, cellular senescence and apoptosis. We contrast rodent and human data, where MAM composition and glucotoxic responses diverge, and consider therapeutic strategies, such as incretin-based agents and the mitochondrial-derived peptide MOTS-c, that target this interface to preserve β-cell function in diabetes.

## 1. Introduction

Eukaryotic cells contain several membrane-bound organelles that occupy specific locations within the cell and possess unique functions. Organelle–organelle communications form an interactive network to coordinate and facilitate cellular function and maintain homeostasis within the cellular environment. Recent studies have reported the existence of membrane contact sites, as adjudged by the close proximity of membranes (10–30 nm distance) between two different organelles [1,2].

From these studies, the endoplasmic reticulum (ER) has been shown to have the most interactions, and with almost all membrane-bound organelles including mitochondria. The ER coordinates protein synthesis, folding and trafficking while maintaining intracellular calcium (Ca^2+^) homeostasis and lipid metabolism [3,4,5]. To preserve homeostasis within the ER when facing dysfunction linked to the formation and folding of secretory proteins, cells engage the intracellular signaling pathway known as the unfolded protein response (UPR) [6]. In individuals with type 2 diabetes (T2D), pancreatic β-cells have certain ER stress indicators suggesting heightened UPR activation [7,8]. Pancreatic β-cells use this UPR signaling to regulate cellular homeostasis in response to protein misfolding stress, but a chronic or excessive ER stress response can trigger apoptosis [7]. Accumulating evidence now shows that ER stress can contribute to the pathogenesis of T2D, contributing to pancreatic β-cell loss and insulin resistance [9].

Mitochondria are crucial organelles in eukaryotic organisms that play a central role in cellular energy metabolism. Mitochondria regulate lipid and glucose metabolism and generate adenosine triphosphate (ATP) by utilizing the energy released through oxidative phosphorylation (OXPHOS) and β-oxidation of fatty acids [10]. In pancreatic β-cells, mitochondrial dysfunction has been linked with diabetes since mitochondrial dysfunction leads to β-cell dysfunction and insulin resistance [11,12]. Mitochondria are one of the main sources of reactive oxygen species (ROS) and also mediate cellular apoptosis and cell death [13]. Excessive ROS production can impair mitochondrial function, and this can be exacerbated by hyperglycemia [14]. Moreover, an increased level of glucose, as occurs following food ingestion, induces OXPHOS in β-cells, which raises the ATP/adenosine diphosphate (ADP) ratio and intracellular Ca^2+^ level inside the cells, leading to the release of insulin. β-cells from patients with T2D exhibit mitochondrial swelling and decreased ATP levels in addition to the elevated circulatory levels of insulin [15].

Contact sites between the ER and mitochondria play a major role in pancreatic β-cell dysfunction and the development of T2D. The reported “physiological” gap between the ER and the outer mitochondrial membrane is approximately 10 to 80 nanometers [16]. The direct communication between the ER and mitochondria occurs through specialized structural regions called mitochondria-associated membranes (MAMs), which enable the transfer of Ca^2+^ and lipids between the two organelles and serve as sites for cellular signaling [17]. The two organelles are physically linked at MAMs through proteins and protein complexes [18]. Malfunction in ER–mitochondria communication can lead to age-related diseases such as neurodegenerative diseases, cardiovascular diseases and cancer [17,19,20]. This review aims to analyze current knowledge about the effects of ER–mitochondria coupling on critical stress factors, including oxidative stress, ER stress, autophagy, mitophagy and cellular senescence that both individually and in concert induce diabetes pathogenesis.

While this review concentrates primarily on T2D, the ER–mitochondria interface also contributes to β-cell destruction in type 1 diabetes (T1D). In T1D, pro-inflammatory cytokines, predominantly interleukin-1β (IL-1β), tumor necrosis factor-α (TNF-α) and interferon-γ (IFN-γ), released during insulitis, trigger concurrent ER stress and mitochondrial dysfunction in β-cells. At the ER–mitochondria interface, these inflammatory signals disrupt Ca^2+^ homeostasis, amplify mitochondrial ROS production, and promote generation of modified autoantigens that autoreactive CD8^+^ T cells recognize [21]. Both organelles function as co-regulators of β-cell immune visibility, not merely as sequential effectors of inflammatory injury. This positions the MAM as a shared mechanistic node across both major diabetes subtypes, though the triggers and downstream consequences differ substantially.

## 2. Cellular Stresses in Pancreatic β-Cells Involving ER and Mitochondria

Pancreatic β-cells are highly specialized insulin-secreting cells that are particularly vulnerable to a variety of cellular stresses due to their high metabolic rate and secretory demand. These stresses include oxidative stress, ER stress, mitochondrial dysfunction, lipotoxicity, hypoxia and inflammatory stress, all of which contribute to β-cell dysfunction and death.

### 2.1. Endoplasmic Reticulum Stress

β-cells dedicate much of their biosynthetic capacity to insulin production, so they rely heavily on ER proteostasis [22]. When unfolded or misfolded proteins accumulate, the UPR is activated to temper protein synthesis, aid folding and trafficking, and target misfolded proteins for degradation; once these adaptive responses are overwhelmed, β-cells undergo apoptosis, and the resulting cell loss drives the functional decline characteristic of T2D [23,24,25] (Figure 1).

### 2.2. Oxidative Stress

The oxidative stress response is also important in T2D pathogenesis. Many studies have reported a relationship between T2D and oxidative stress both in vitro and in mouse models [26,27]. The insulin signaling pathway is linked to free radical production and activation [26,27]. Increased production of free radicals in T2D is due to increased non-enzymatic glycation of proteins, increased lipid peroxidation and glucose oxidation; free radicals provoke dysregulation of enzymes and cellular organelles [26,27,28]. Inherently, β-cells have reduced antioxidant protection and, together with increased free radical production in T2D, β-cell loss can result [26,27,28]. ROS and insulin are also closely interconnected, with insulin stimulating ROS production; however, ROS negatively impact insulin signaling [26,27,28], the impact at target tissues being due to activation of pathways such as p38 mitogen-activated protein kinase, NH_2_-terminal Jun kinases/stress-activated protein kinases and nuclear factor-κB [28]. Each of these pathways is stress sensitive and is upregulated in T2D, increasing insulin resistance and further impairing β-cell function (Figure 2).

The reactive oxygen species generated through disturbed insulin signaling and glucose oxidation are the same species that subsequently act at MAM-associated redox nanodomains, a convergence developed in Section 4.1. Beyond these signaling cascades, oxidative stress compromises the transcriptional machinery that maintains β-cell identity. The insulin promoter transcription factors PDX1 (pancreatic and duodenal homeobox-1) and MafA are exquisitely sensitive to chronic hyperglycemia and ROS: their nuclear expression and DNA binding activity decline in diabetic islets, suppressing insulin gene transcription, and antioxidant treatment restores both factors together with insulin content [29,30,31]. In parallel, oxidative and metabolic stress drives nuclear relocation of the forkhead factor FOXO1; in primary human β-cells, hydrogen peroxide shuttles FOXO1 to the nucleus and provokes loss of MAFA and PDX1 with acquisition of progenitor markers, indicating that ROS can precipitate a partial loss of β-cell identity (dedifferentiation) rather than only apoptosis [32,33]. This transcription factor axis links the oxidative environment directly to the progressive functional decline and identity loss that characterize β-cell failure in T2D (Figure 2).

### 2.3. Autophagy and Mitophagy

Autophagy sustains β-cell survival by clearing misfolded proteins and damaged organelles and is essential for maintaining β-cell mass and insulin secretory function under nutrient, hypoxic and ER stress [34,35,36]. Two seminal in vivo studies first established this: β-cell-specific autophagy-related 7 (Atg7) deletion caused islet degeneration, reduced β-cell mass and impaired glucose tolerance [37], while high-fat feeding induced a compensatory increase in β-cell autophagy whose loss precipitated glucose intolerance [38]. Autophagy is chiefly controlled by mTORC1, inhibited during fasting to permit autophagy and activated on refeeding to favor insulin secretion [39]; both loss of mTORC1 and its chronic hyperactivation disrupt β-cell homeostasis [34]. Autophagy additionally governs insulin homeostasis by degrading excess proinsulin at the trans-Golgi network, preventing Golgi overload; its inhibition (Atg5/Atg7 knockdown or bafilomycin A1) causes accumulation of proinsulin and chromogranin A and dysregulated glucose-stimulated secretion [40].

Mitophagy selectively removes damaged mitochondria to preserve β-cell function under inflammatory, oxidative and metabolic stress [41,42] and is orchestrated by TFEB, CLEC16A, PHB2 and the Nrf2/PHB2 and PTEN-induced kinase 1 (PINK1)/Parkin pathways [42]. TFEB-mediated mitophagy, triggered by lysosomal Ca^2+^ release, preserves glucose tolerance during high-fat feeding [43]; CLEC16A counteracts cytokine-induced β-cell apoptosis [41,44]; and PHB2 protects mitochondria via the Nrf2/PHB2 pathway [42]. Several agents (IL-6, nobiletin, cyanidin-3-O-glucoside, silybin) promote protective mitophagy [45,46,47,48], and sonodynamic activation of PINK1/Parkin mitophagy relieves lipotoxic β-cell exhaustion [49], whereas long-term impairment of mitophagy compromises β-cell differentiation, survival and growth [50,51]. The mechanisms by which ER–mitochondria contacts regulate autophagy and mitophagy are examined in detail in Section 4.2 and Section 4.3.

## 3. The Role of ER–Mitochondria Interaction in Health in β-Cells

Having outlined the individual stress pathways that threaten β-cell viability, we next consider how physical and functional coupling between the ER and mitochondria normally safeguards β-cell function, before turning to how this crosstalk fails under disease conditions. We begin by surveying the direct evidence that ER–mitochondria contacts exist and are dynamically regulated in β-cells, because such studies remain scarce, and much of the current framework rests on the behavior of MAM-associated proteins and on findings extrapolated from non-β-cell systems.

### 3.1. Mitochondria-Associated Membranes (MAMs)

Mitochondria-associated membranes (MAMs) are specialized subdomains of the ER that come into close apposition (typically 15–30 nm) with the outer mitochondrial membrane, forming ER–mitochondria contact sites (MERCs). Rather than possessing a unique resident lipid or protein, MAMs are defined by the relative enrichment of a characteristic set of tethering and signaling components [52]. In most cell types, these include the inositol 1,4,5-trisphosphate receptor (IP3R)–glucose-regulated protein 75 (GRP75)–voltage-dependent anion channel 1 (VDAC1) Ca^2+^-transfer complex, mitofusin 2 (Mfn2), phosphofurin acidic cluster sorting protein 2 (PACS-2), the VAPB–PTPIP51 tether and PDZ domain-containing protein 8 (PDZD8), together with resident chaperones and the ER stress sensors that dock at the interface. For clarity, throughout this review, the term MAMs denotes the biochemically defined, protein- and lipid-enriched ER subdomain that becomes apposed to mitochondria, whereas MERCs (ER–mitochondria contact sites) denotes the physical membrane contact itself; although the two terms are frequently used interchangeably in the literature, they are applied in this consistent sense throughout the manuscript.

### 3.2. Direct Evidence for ER–Mitochondria Contacts in β-Cells: State of the Evidence

Compared with liver, muscle and neuronal systems, very few studies have directly examined ER–mitochondria contact sites in pancreatic β-cells; recent evaluations of the field note that very few studies have directly investigated the role of MAMs in β-cells [53] and that the role of MAMs in β-cells is still largely unknown [54]. Consequently, many statements about ER–mitochondria crosstalk in β-cells rest on quantification of MAM-enriched proteins rather than of contacts themselves, and on functional extrapolation from other cell types, whose metabolic and Ca^2+^-handling properties differ substantially from those of β-cells [21]. The most direct evidence in human tissue comes from in situ proximity ligation assays. Thivolet et al. examined pancreatic specimens from 12 donors with type 2 diabetes and nine non-diabetic controls obtained through the Network for Pancreatic Organ Donors with Diabetes (nPOD), using immunofluorescence, in situ hybridization and in situ proximity ligation to quantify ER–mitochondria interconnection through MAMs [55]; ER–mitochondria interactions measured in this way are reduced in β-cells from T2D donors compared with non-diabetic controls [53,55]. Consistently, the number of IP3R–VDAC1 complexes assessed by in situ proximity ligation in pancreatic tissue sections is significantly lower in β-cells from individuals with T2D than in non-diabetic controls [21]. Direct functional interrogation of contacts in β-cell models is similarly limited. In INS-1E cells, acute glucose stimulation increased both ER–mitochondria interactions and ER–mitochondria Ca^2+^ exchange, whereas experimental disruption of MAMs altered glucose-stimulated insulin secretion; conversely, chronic high glucose in INS-1E cells and human islets reduced the ER Ca^2+^ store, raised basal mitochondrial Ca^2+^, and reduced ATP-stimulated ER–mitochondria Ca^2+^ exchange despite an increase in organelle interactions [54]. Glucose also stimulates MAM formation in INS1E β-cells, MAM disruption inhibits glucose-stimulated insulin secretion (GSIS), and chronic glucotoxicity induces MAM formation in human islets [54], while palmitate treatment of MIN6-β cells altered both GSIS and ER–mitochondria interactions [53]. In addition, a recent study in INS-1E cells demonstrated that dysregulated MAMs constitute a mechanistic link between saturated FFA-induced lipotoxic stress and β-cell failure in T2D [56]. At the channel level in primary β-cells, a continuous ER Ca^2+^ leak directed to mitochondria, established by glycogen synthase kinase 3β (GSK3β)-mediated phosphorylation of presenilin-1, increases basal respiration and organellar ATP [57], and glucose-triggered cytosolic Ca^2+^ spiking together with initial, but not long-lasting, insulin secretion in pancreatic islets depends on this ER Ca^2+^ leak [58]. Finally, tether-related proteins with demonstrated β-cell roles include junctophilin-3, expressed in mouse and human β-cells, whose knockdown disturbs ER–mitochondria spatial organization and impairs GSIS [59], and PDZD8, which augments ER–mitochondria contact and mitochondrial Ca^2+^ flow in β-cells during diabetes [60]. These studies define the boundaries of what is currently demonstrated in β-cells; wherever the remainder of this section draws on other cell types, this is stated explicitly.

### 3.3. ER Ca^2+^ Handling in β-Cell Physiology and Its Perturbation in Diabetes

Ca^2+^ homeostasis is central to β-cell function because cytosolic Ca^2+^ is the proximal trigger of exocytosis and because organelle Ca^2+^ uptake fine-tunes both cytosolic dynamics and organellar function. Glucose raises ATP, closes K-ATP channels and depolarizes the plasma membrane, opening voltage-gated Ca^2+^ channels; the resulting cytosolic Ca^2+^ signal is rapidly reshaped by sarco/endoplasmic reticulum Ca^2+^-ATPase (SERCA)-mediated ER uptake, such that [Ca^2+^]c and [Ca^2+^]ER oscillate in phase during glucose stimulation, with Ca^2+^ taken up by SERCA2b and SERCA3 and, when Ca^2+^ influx is strong, released from the ER in a SERCA3-dependent manner [61,62]. During each cytosolic Ca^2+^ pulse, mitochondria, ER, secretory granules and lysosomes take up Ca^2+^, fine-tuning cytosolic dynamics and controlling organellar function [63]. Mitochondrial Ca^2+^ follows the cytosolic signal, and this coupling is functionally important: silencing the mitochondrial calcium uniporter (MCU), or retarding the mitochondrial Ca^2+^ rise, attenuates ATP synthesis and second-phase insulin secretion [64], and feedback from oscillatory cytosolic Ca^2+^ controls mitochondrial ATP generation. ER Ca^2+^ mobilization itself contributes primarily to sustaining, rather than initiating, glucose-induced Ca^2+^ waves [65]. Mitochondria are additionally positioned close to plasma membrane Ca^2+^ channels, where they sense high-Ca^2+^ microdomains and convert Ca^2+^ uptake into ATP that fuels exocytosis [66]. Within this framework, the ER–mitochondria interface is the route by which the ER Ca^2+^ store can feed mitochondrial metabolism independently of, and in addition to, plasma membrane Ca^2+^ influx. In β-cells, this route is physiologically engaged: the continuous ER Ca^2+^ leak directed to mitochondria via GSK3β-phosphorylated presenilin-1 raises basal respiration and organellar ATP [57], enhancing mitochondrial responsiveness during the first phase of elevated glucose and thereby GSIS [67], and acute glucose strengthens ER–mitochondria Ca^2+^ exchange in INS-1E cells, with MAM disruption altering GSIS [54]. The Ca^2+^ abnormalities recorded in diabetic models, summarized below, should therefore be read not only as defects of ER Ca^2+^ storage per se, but also as potential failures of ER-to-mitochondria Ca^2+^ transfer.

A comparative study of islets from Goto–Kakizaki (GK) and neonatal streptozotocin (nSTZ) rats, two well-established models of non-insulin-dependent diabetes mellitus (NIDDM), revealed several common dysfunctions of [Ca^2+^]i handling in response to glucose, thus indicating a role in the poor insulin secretion observed in these animals [68], suggesting that Ca^2+^ metabolism may be the common abnormality between different forms of diabetes [69]. In 2009, Lim et al. demonstrated that mitochondrial dysfunction triggers the ER stress response in human liver SK-HepI cells, leading to elevation of cytosolic free Ca^2+^ and activation of p38 mitogen-activated protein kinase (MAPK) [70]. This ER stress response can cause abnormal insulin signaling and increased hepatic gluconeogenesis. Diabetic mouse models demonstrate deficient Ca^2+^ oscillations following glucose stimulation [71,72]. Studies in human islets indicate that abnormalities in glucose-stimulated Ca^2+^ can lead to T2D development [73]. Under chronic hyperglycemia, elements of the Ca^2+^ signaling cascade, such as the Ca^2+^-sensitive protein phosphatase calcineurin (CaN) and Calcium/calmodulin-stimulated protein kinase II (CAMKII), are overstimulated, leading to β-cell dysfunction [74,75]. This is partly due to loss of the SERCA protein in T2D islets, and this reduction leads to ER stress and β-cell death [72]. Ca^2+^ is released from the ER by activating IP3R, but this IP3R-mediated release is reduced under ER stress conditions [76], suggesting that β-cell ER stress is regulated by Ca^2+^ channels in the ER. Ca^2+^ in different cell compartments must be tightly regulated to maintain homeostasis. ER Ca^2+^ depletion with subsequent increase in cytoplasmic Ca^2+^ occurs in oxidative stress, ER stress and chronic hyperglycemia, and can trigger β-cell death [77].

Notably, chronic hyperglycemia has now been shown to deplete the ER Ca^2+^ store and to reduce ATP-stimulated ER–mitochondria Ca^2+^ exchange despite increased organelle contacts, in both INS-1E cells and human islets, a direct mechanistic bridge between the diabetic Ca^2+^ phenotype and ER–mitochondria crosstalk. The loss of SERCA protein in T2D islets (above) plausibly curtails the Ca^2+^ available for transfer to mitochondria, although this prediction remains to be tested directly in β-cells.

### 3.4. Molecular Components of MAM That Are Involved in Intracellular Ca^2+^-Transfer in β-Cells

Mitochondria and ER are critical organelles involved in transfer, storage and regulation of Ca^2+^. Ca^2+^ concentration inside the ER is 1 mM and ~100 nM in the cytosol [78]. MAMs, zones of close contact between ER and mitochondria, play an important role in transmission of Ca^2+^ signals from ER to mitochondria, the optimal distance between membranes being 15–20 nm [16]. Most of the mechanistic detail of the IP3R–VDAC–GRP75–MCU axis (including the transfer of Ca^2+^ from IP3Rs to mitochondria in high-Ca^2+^ microdomains upon histamine stimulation, and the chaperone-mediated coupling of the two channels) derives from non-β-cell systems and is presented here as the general framework. Whether each element operates identically in β-cells, whose physiological stimuli are glucose- and incretin-driven rather than histamine-driven, is largely untested. Rapizzi et al. reported voltage-dependent anion channels (VDAC) at mitochondria (MT)–ER contact (MERC) sites, which facilitate mitochondrial Ca^2+^ accumulation by allowing Ca^2+^ to rapidly diffuse from ER to the inner mitochondrial membrane [79]. β-cells express two isoforms of VDAC (VDAC1 and VDAC2) [80,81]. In 2017, Thivolet et al. reported that mitochondrial VDAC1 protein expression in T2D β-cells was reduced despite increased expression of tethering proteins, indicating that T2D β-cells have reduced ER–mitochondria interactions [55]. By contrast, in 2019, Zhang et al. reported that hyperglycemia increases VDAC1 gene expression in islets from T2D and non-diabetic organ donors under glucotoxic conditions and that VDAC1 upregulation leads to ATP depletion and impaired insulin secretion [81]. It is worth being explicit about the limits of these VDAC1 data: the two reports are discordant, and neither directly quantified ER–mitochondria contacts, so both are consistent with, but do not demonstrate, altered ER–mitochondria interaction. By contrast, direct assays of contact abundance and exchange in β-cell models show that acute glucose strengthens ER–mitochondria Ca^2+^ exchange and that chronic glucotoxicity disrupts it despite increased contacts [54]. These findings caution against equating altered tethering protein abundance with altered functional coupling.

These results indicate the role of altered VDAC gene expression and protein localization in mitochondrial dysfunction. Interestingly, the accumulation of Bcl-2, an antiapoptotic protein, on MAMs has been reported, thus playing a role in Ca^2+^ homeostasis since excess Ca^2+^ within cells is highly toxic [82]. It has been proposed that at mitochondria-associated ER membranes (MAMs), Bcl-2 fine-tunes intracellular Ca^2+^ homeostasis by directly binding to IP_3_ receptors, suppressing pro-apoptotic ER-to-mitochondria Ca^2+^ transfer while sustaining basal flux essential for cellular bioenergetics. This localized regulation prevents toxic mitochondrial Ca^2+^ overload and regulates bioenergetic microdomains to suppress apoptosis and maintain cell survival [83]. However, more research on the targeting mechanisms of the Bcl-2 family proteins in mitochondria is needed.

Upon cell stimulation with histamine, mitochondria can directly take up Ca^2+^ from IP3Rs, microdomains of high intracellular Ca^2+^ that can be transferred from ER to mitochondria [84]. Stimulation by hyperglycemia can impair mitochondrial Ca^2+^ uptake and release [85]. An in vivo study found that a high-fat, high-sucrose diet in mice reduced IP3-stimulated Ca^2+^ transfer to mitochondria. IP3R and VDAC channels at ER and the outer mitochondrial membrane allow for transfer of Ca^2+^ (Figure 3) [15,86]. Both these channels are regulated by the chaperone protein GRP75 [86]. Ca^2+^ is transported to the inner mitochondrial membrane by the MCU [86].

There are many proteins present in the MAM complex that modulate Ca^2+^ signaling in the microdomains as well as stabilizing ER–mitochondria tethering by regulating the folding of newly synthesized proteins, mitochondria-localized dehydrogenase activity and activation of enzymes that control apoptosis. Proteins such as calnexin (CNX) and calreticulin are present on MAMs and regulate Ca^2+^ flux from ER to mitochondria by interacting with IP3Rs and SERCAs [86]. If MAM proteins are overexpressed, excess Ca^2+^ storage in mitochondria can occur, leading to ER stress response-mediated apoptosis [86]. This mechanism has been studied in β-cells, though the literature is conflicting. A phenotypic study of compounds that protect β-cells from glucolipotoxicity indicated that Ca^2+^ overload in pancreatic islets was the main mechanism of glucolipotoxicity-mediated apoptosis and dysfunction [92]. Moreover, a study on mouse clonal pancreatic β-cells showed that ER Ca^2+^ depletion, induced by palmitate, led to ER stress and cell death [93]. However, Weiser et al. found that glucose stimulation in a pancreatic β-cell model leads to increased mitochondrial Ca^2+^ uptake, thus playing a key role in the metabolic coupling of insulin [94]. This was consistent with other studies that found that suppression of mitochondrial Ca^2+^ increase leads to reduced glucose-induced respiration, ATP synthesis, and impairment of second-phase insulin secretion [94,95]. Thus, the effects of Ca^2+^ overload in the mitochondria of β-cells remain to be clarified. These principal regulators, their localization, proposed function, effect on Ca^2+^ and the level of direct β-cell evidence are summarized in Table 1.

### 3.5. Mitofusin 2 (Mfn2) in Regulating ER-MAM Interactions

Mfn2 is a key modulator of the UPR and suppresses protein kinase R-like ER kinase (PERK), an ER stress sensor; Mfn2 is an established ER–mitochondria tether. Loss of Mfn2 leads to ER stress [99,100]. In Ca^2+^ transport, Mfn2 on the ER surface interacts with Mfn1 and Mfn2 on the mitochondrial membrane [86]. An in vivo study on neuronal cells found that removal of Mfn2 resulted in reduced close contact between ER and mitochondria whereas Mfn2 overexpression caused increased close contacts [101]. Mitofusins (Mfn1/2) are essential for maintaining normal glucose-stimulated calcium dynamics, mitochondrial membrane potential, and ATP levels in β-cells. In β-cells, the deletion of Mfn1/2 leads to β-cell loss and disrupts cell-to-cell connectivity. This disruption significantly alters mitochondrial morphology, resulting in smaller, rounder organelles with reduced elongation and perimeter in βMfn1/2-KO cells. Furthermore, β-cells in double knockout (dKO) mice display defective insulin secretion despite retaining a pool of morphologically docked granules [102]. Mitochondrial Ca^2+^ uptake and IP3R-Grp75 interaction was also reduced following Mfn2 ablation but increased following Mfn2 overexpression [101], highlighting the role of Mfn2 in ER–mitochondria tethering and Ca^2+^ transfer. However, ER Ca^2+^ mobilization remains unaffected in dKO islets; rather, they exhibit decreased mitochondrial oxygen consumption (VO_2_) and mtDNA depletion [102]. Furthermore, Gottschalk et al. found that Mfn2 mediated a longer lifetime and increased stability of MAMs during ER stress; ER stress was followed by increased ER Ca^2+^ leak, stable MAMs, and increased mitochondrial Ca^2+^ uptake causing increased tricarboxylic acid (TCA) cycle activity and mitochondrial membrane potential, oxidative phosphorylation and ATP generation [103]. ATP production was driven by oxidative phosphorylation and moved towards the ER. Mfn2 played a critical role in MAM stability, ATP transport into the ER lumen, and transport of Ca^2+^ into mitochondria [103].

The interpretation of Mfn2 as an ER–mitochondria tether deserves an explicit caveat. Although Mfn2 was originally proposed as a physical tether between the two organelles, quantitative electron microscopic analysis in non-β-cell systems showed that Mfn2 ablation increases, rather than decreases, structural and functional ER–mitochondria coupling, giving rise to an alternative model in which Mfn2 acts as a “tethering antagonist” preventing excessive proximity [89]. A subsequent critical reappraisal using electron microscopy and FRET-based proximity probes reaffirmed that constitutive or acute Mfn2 deletion reduces organelle juxtaposition and mitochondrial uptake of ER-released Ca^2+^, supporting Mfn2 as a bona fide tether [90], and the recent identification of the ER-specific MFN2 splice variant ERMIT2 (which localizes to the ER–mitochondria interface, tethers the two organelles, and enables efficient mitochondrial Ca^2+^ uptake and phospholipid transfer) adds a further layer of complexity [89,90]. This debate has been conducted in fibroblasts, neurons and liver [104], not β-cells. Accordingly, the consequences of mitofusin loss reported in βMfn1/2 dKO islets (fragmented mitochondria, reduced ATP, impaired Ca^2+^ responses and GSIS) do not by themselves demonstrate that ER–mitochondria tethering is altered in these cells; direct measurement of contact architecture in mitofusin-deficient β-cells is awaited.

### 3.6. ER Stress Sensors and MAM Proteins: Regulation of UPR

This subsection separates two distinct roles of IRE1α that are often conflated: its demonstrated functions in β-cell UPR signaling and insulin biosynthesis, and its non-canonical, MAM-localized functions, which have so far been established only in non-β-cell systems. In β-cells, IRE1α is activated by high glucose in pancreatic islets and supports proinsulin translation and insulin content, whereas its chronic activation or deletion has context-dependent outcomes.

Besides MAM proteins, ER stress sensor proteins are also present at the MAM and play a role in ER–mitochondria communication. These sensors act independently to induce the UPR and maintain ER homeostasis. Of note are inositol-requiring enzyme 1 (IRE1) and PERK. IRE1 is present in two kinase forms: IRE1α and IRE1β. IRE1α is present widely throughout various tissues, whereas IRE1β is limited to the cells lining the intestinal epithelium. High glucose activates IRE1α in pancreatic islets [105]. Importantly, the localization of IRE1α to MAMs enables ER stress signaling to be directly integrated with mitochondrial metabolism, calcium handling, and apoptotic sensitivity.

In this context, the activation of IRE1α by insulin secretagogues differs from its activation during ER stress. Knocking down IRE1 signaling reduces insulin biosynthesis, while prolonged activation of IRE1 signaling leads to a decrease in insulin gene expression [96]. Deletion of IRE1α in adult β-cells in mice reduced proinsulin translation, resulting in decreased pancreatic proinsulin and insulin content as well as impaired postprandial insulin secretion, accompanied by reduced mRNA levels of proteins involved in ribosome recruitment to the ER and in mRNA processing. Notably, β-cell-specific deletion of IRE1α in non-obese diabetic (NOD) mice has been reported to confer protection against autoimmunity through a mechanism involving β-cell dedifferentiation, suggesting that loss of IRE1α in β-cells has remarkably different outcomes depending on the cellular stress context [96].

Mechanistically, IRE1α activates multiple downstream pathways via both its kinase and endonuclease functions following ER stress [106]. Upon stress sensing, the UPR pathway is initiated through IRE1α oligomerization or dimerization, ultimately leading to trans-autophosphorylation [106]. IRE1α cleaves the XBP1 intron and generates a stable transcription element, XBP1s, which promotes protein folding during ER stress, maintains β-cell identity, and prevents the onset of diabetes in insulin-resistant states [107,108]. However, chronic or unresolved activation of IRE1α can shift signaling towards apoptosis.

Protein Kinase B-mechanistic target of Rapamycin (PKB/AKT-mTOR) has been shown to modulate IRE1 deactivation by regulating ER–mitochondria communication at MAMs [109]. Deactivation occurs through a two-step process, involving the initial termination of IRE1 RNAse activity through autophosphorylation, followed by complete silencing only after ER–mitochondrial contacts are re-established following stress-induced uncoupling. One proposed mechanism is that AKT-mTOR-dependent stabilization of MAMs alters the lipid environment, particularly cholesterol enrichment, thus modifying IRE1 activation and deactivation thresholds [109]. The mechanisms underlying this signaling in pancreatic β-cells, and whether it represents a therapeutic target in disorders such as T2D characterized by dysregulated AKT-mTOR signaling and abnormal UPR activation, remain to be fully defined.

Beyond IRE1’s canonical role in the UPR, it can also contribute to the maintenance of MAM composition and function. Carreras-Sureda et al. proposed that IRE1α stabilizes inositol-1,4,5-trisphosphate (InsP3) receptors (InsP3Rs) at the MAM and controls their distribution at MAMs by acting as a “scaffold” [110]. This MAM-localized role of IRE1α (acting as a scaffold that stabilizes InsP3Rs at MAMs, promotes mitochondrial Ca^2+^ uptake, and influences mitochondrial respiration independently of its UPR function) has been demonstrated in non-β-cell lines, where genetic strategies separated these effects from canonical UPR signaling [110]; ER stress sensors more generally are recognized to localize at MAMs and to function beyond ER stress [86]. It remains untested whether IRE1α localizes to MAMs in β-cells or performs this scaffolding role in the islet: the β-cell data currently available address canonical IRE1α signaling, not its MAM-localized functions. This greatly affects the formation of the protein complex between IRE1α and InsP3Rs, which can stimulate Ca^2+^ uptake by mitochondria. The study also found that IRE1α-deficient cells had a greater distance between ER and mitochondria, impairing Ca^2+^ transfer into mitochondria. Moreover, IRE1α was found to mediate ER–mitochondria communication in the absence of ER stress as well as influence mitochondrial bioenergetics such as ATP production and phosphorylation of AMP-activated protein kinase (AMPK) in the context of ER stress. This suggests the possibility for therapeutic interventions that target the bioenergetic pathways of ER stress for treatment of diseases involving dysfunctional MAMs. In this context, InsP3 can release Ca^2+^ from a nonmitochondrial pool in RIN-m5F cells. The accumulation of InsP3 precedes both the rise in intracellular Ca^2+^ and the peak in insulin secretion, and its addition to the microsomal fraction from RIN-m5F cells evoked a rapid Ca^2+^ release [111]. InsP3 is able to evoke this response by binding to a cytoplasmic receptor located in the ER, the InsP3R. Once activated, the InsP3R releases stored Ca^2+^ [112]. In glucose-stimulated β-cells, this pathway is coupled to membrane excitability: Ca^2+^ mobilized from InsP3-sensitive stores, an effect that can also be evoked by intracellular GTP, drives oscillatory electrical activity that parallels the pulsatile release of insulin, reflecting a close interplay between plasma membrane ion channels and InsP3-induced Ca^2+^ mobilization [113]. Consistent with a regulatory role for this second messenger, β-cells lacking the Ca^2+^-channel β3 subunit show enhanced InsP3 formation and greater Ca^2+^ mobilization from intracellular stores; the β3 subunit therefore negatively modulates InsP3-induced Ca^2+^ release without affecting the voltage-gated L-type Ca^2+^ channel [114]. Extracellular Ca^2+^ does not further raise [Ca^2+^]i once the InsP3-sensitive pool has been emptied by carbamylcholine stimulation, indicating that both stimuli draw on the same pool; the accompanying pronounced insulin release suggests that the Ca^2+^-induced rise in [Ca^2+^]i is itself mediated through InsP3 formation [115]. Ca^2+^ released by InsP3 is subsequently taken up by an InsP3-insensitive region of the ER and, upon InsP3 metabolism, recycles back to the high-affinity InsP3-sensitive pool independently of mitochondria; thus, although the ER is central to Ca^2+^ buffering in β-cells, InsP3 modulates Ca^2+^ transport only transiently before the InsP3-insensitive ER resumes continuous regulation of Ca^2+^ cycling [116].

### 3.7. The Role of PERK in Maintaining ER-MAM Morphology

As with IRE1α, PERK’s roles in β-cells must be separated from its MAM-localized functions demonstrated in other cell types. In β-cells, the demonstrated actions of PERK are its canonical UPR functions (eIF2α phosphorylation, ATF4 activation, regulation of proinsulin proteostasis via ER chaperones such as BiP and ERp72), together with the coupling between falling glucose, reduced [Ca^2+^]ER and PERK–eIF2α activation described below, and the enhancement of GSIS by partial PERK inhibition.

PERK is another ER stress sensor that becomes activated through its dimerization and autophosphorylation during ER stress (Figure 4). Activation of PERK leads to phosphorylation of eukaryotic initiation factor 2 alpha (eIF2α), which modulates misfolded protein overload and inhibits mRNA translation [117]. Phosphorylation of eIF2α also allows for the activation of activating transcription factor 4 (ATF4), which regulates protein folding, autophagy and apoptosis [98]. Interestingly, partial inhibition of PERK activity increased GSIS by enhancing cytosolic Ca^2+^ transit in mouse and human islets, suggesting the role of PERK in pancreatic β-cell function [97]. Verfaillie et al. demonstrated that PERK at MAMs is essential for maintaining ER morphology and Ca^2+^ signaling [118].

Early in ER stress, PERK covalently interacts with the oxidoreductase ERO1α, and this complex increases MERCs, preventing energy depletion and oxidative stress [120]. The complex also facilitates transfer of Ca^2+^ between ER and mitochondria to increase mitochondrial fitness and prevent cell death during ER stress [120]. However, in β-cells, a decrease in glucose concentration leads to a concentration-dependent reduction in [Ca^2+^] ER, which parallels the activation of PERK and the phosphorylation of its substrate, eIF2α. This reduction in [Ca^2+^]ER is attributed to decreased activity of the sarcoplasmic/ER Ca^2+^-ATPase pump, mediated by a decline in the energy status of the cell [121]. Balsa et al. reported that ER stress and glucose deprivation stimulate mitochondrial bioenergetics by forming respiratory super complexes (SCs) in human osteosarcoma (U2OS) cells [119]. This increase in SCs is mediated by PERK, thereby increasing ATP production and promoting proteostasis [119]. In β-cells, PERK plays a role in regulating proinsulin proteostasis by modulating ER chaperones such as BiP and ERp72 [122]. This indicates a distinct mechanism of PERK-mediated proteostasis that may operate independently of mitochondrial super complexes. Beyond PERK’s role in stressed conditions, a recent study explored PERK’s ability to regulate homeostatic lipid trafficking and found that it promotes lipid transfer between ER and mitochondria in unstressed cells to encourage mitochondrial homeostasis and respiration [123]. PERK’s role in maintaining mitochondrial homeostasis aligns with findings in β-cells, where a reduction in PERK levels (induced by palmitate) was associated with decreased mitochondrial volume density in human islets [124]. PERK is additionally one of the ER stress sensors enriched at MAMs: PERK-deficient cells show a decreased number of ER–mitochondria contact sites and perturbed ER Ca^2+^ signaling [118], and PERK localized at ER–mitochondria contact sites facilitates the propagation of ROS-based apoptotic signals from the ER, a function retained by a kinase-dead PERK mutant, indicating tethering independent of UPR transducer activity [125,126]. During ER stress, PERK interacts covalently with ERO1α to promote oxidation of contact site proteins and improve ER–mitochondria Ca^2+^ flux while limiting oxidative stress [120], forms mitochondria–ER contact sites through interaction with ATAD3A that protect local mitochondrial translation [127], and drives protective stress-induced mitochondrial hyperfusion via eIF2α-dependent translation attenuation [128], in keeping with a broader PERK–mitochondria axis at contact sites [129]. These mechanisms, established in non-β-cell lines, provide a plausible template for how ER stress could be communicated to mitochondria in β-cells; whether MAM-localized PERK performs them in islets is currently untested, since the β-cell-specific PERK data concern canonical UPR signaling and proteostasis, not contact site biology. These studies strongly imply the role of PERK in modulating ER–mitochondria communication during ER stress as well as in non-stress conditions; however, the complete mechanism in pancreatic β-cells remains to be elucidated.

### 3.8. ER–Mitochondria Kinetics

In β-cells, the studies with the most direct bearing on contact architecture are those summarized in Section 3.2 and Section 3.4. ER–mitochondria kinetics are dynamically regulated in response to cellular stress and are particularly relevant in pancreatic β-cells, which rely on tightly coordinated organelle communication to sustain insulin biosynthesis and secretion. Earlier experimental work in non-β-cell systems showed that induction of ER stress, including by agents such as tunicamycin or rapamycin, leads to an increase in ER–mitochondria contacts [130,131].

In β-cells, however, chronic metabolic stress appears to disrupt this adaptive remodeling. Human islets from individuals with T2D display altered expression of key ER–mitochondrial tethering proteins, including increased IP3 receptor expression but reduced VDAC1 levels, resulting in fewer functional ER–mitochondria contacts [55]. These structural changes are associated with impaired insulin secretion. Consistent with this, junctophilin-3 (JPH3) has been shown to be expressed in human and mouse β-cells and to support glucose-stimulated insulin secretion by maintaining ER–mitochondria spatial organization and Ca^2+^ signal amplification [59]. Reduced JPH3 expression disrupts ER–mitochondria coupling, impairs mitochondrial function and blunts Ca^2+^-dependent insulin secretion in mouse islets [59].

A recent study by Liu et al. also identifies PDZD8 as an important regulator of ER–mitochondria coupling in β-cells [60]. Under diabetic conditions, increased PDZD8 expression promotes excessive ER–mitochondria contacts and mitochondrial Ca^2+^ overload, contributing to mitochondrial dysfunction, ER stress and β-cell apoptosis [60]. In contrast, limiting PDZD8-mediated contacts alleviates organelle stress and improves β-cell survival [60]. Together, these findings suggest that while transient increases in ER–mitochondria communication may be adaptive, sustained dysregulation of this crosstalk contributes to β-cell failure in T2D.

### 3.9. What Is Demonstrated, Inferred and Hypothetical: Scope and Caveats

The preceding subsections deliberately distinguish three categories of evidence (Table 2). Directly demonstrated in β-cells are reduced ER–mitochondria interaction by in situ proximity ligation in β-cells of T2D donors [21,53,55]; glucose-dependent modulation of ER–mitochondria Ca^2+^ exchange and contact abundance with functional consequences for GSIS in INS-1E cells and human islets [54]; an ER-to-mitochondria Ca^2+^ leak supporting β-cell respiration, ATP and first-phase GSIS [58]; and β-cell roles for JPH3 [59] and PDZD8 [60]. Inferred from other cell types (because the underlying experiments used fibroblasts, hepatocytes, neurons or cell lines) are the IP3R–VDAC–GRP75–MCU transfer mechanism, the Mfn2 tether debate, and the MAM-localized functions of IRE1α and PERK [89,90,110,125]. Remaining hypotheticals in β-cells are whether IRE1α or PERK localize to MAMs in islets; whether mitofusin loss changes contact architecture per se in β-cells; whether MAMs regulate β-cell mitophagy, which is currently unclear; and whether experimental MAM manipulation can restore β-cell function in vivo, for which direct evidence that experimental modulation of MAMs regulates β-cell function is still lacking [53].

Finally, we caution against attributing all disturbances of β-cell autophagy, mitophagy or ER/mitochondrial stress to MAM dysfunction. β-cell mitophagy can proceed or fail independently of MAMs: β-cell-specific Parkin knockout mice showed neither abnormal β-cell function nor altered glucose profiles before or after high-fat feeding, implicating Parkin-independent mitophagy [43]; PINK1 ablation did not affect β-cell mitophagy, glucose tolerance or GSIS in a β-cell mitophagy reporter model in which dysfunctional mitochondria nevertheless accumulated during metabolic stress [132], and it remains unclear whether mitochondrial defects arising from core autophagy loss reflect impaired turnover directly or secondary defects such as ER stress [133]. Altered MAM protein abundance, ER stress and mitophagy changes in T2D β-cells are therefore best viewed as parallel, potentially interacting (but not interchangeable) consequences of metabolic stress.

**Table 2:** Summary of the current evidence for the involvement of ER–mitochondria contact sites in pancreatic β-cell function, classified as directly demonstrated in β-cells, inferred from other cell types, or unresolved/hypothetical.

Topic	Demonstrated in β-Cells	Inferred from Other Cell Types	Unresolved/Hypothetical in β-Cells	References
Contact structure	Reduced in situ PLA signal for ER–mito interaction in T2D donor β-cells	None	Electron microscopic quantification of contact number/distance in β-cells	[21,53,55]
Function in GSIS	Acute glucose strengthens contacts and Ca^2+^ exchange; MAM disruption alters GSIS	IP3R–VDAC–GRP75 microdomain model (existing citations)	Whether contact abundance and exchange competence dissociate under glucotoxicity	[54]
Ca^2+^ transfer	ER → mitochondria presenilin-1/GSK3β leak supports ATP and first-phase GSIS	IP3R–VDAC–GRP75–MCU axis (existing citations)	None	[57,67]
Mfn2	βMfn1/2 dKO: fragmentation, reduced ATP, impaired Ca^2+^/GSIS (existing citation)	Tether role debated: pro-tether vs. “tethering antagonist”	Whether Mfn2 tethers MERCs in β-cells per se	[89,90]
IRE1α	Canonical UPR/insulin biosynthesis roles (existing citations)	Scaffold for InsP3R at MAMs	IRE1α–MAM localization in β-cells	[110]
PERK	eIF2α–ATF4, proinsulin proteostasis, [Ca^2+^]ER coupling (existing citations)	MERC-localized tethering/signaling	MAM-localized PERK function in β-cells	[110,120,125,127]
Mitophagy	Dysfunctional mitochondria accumulate despite mitophagy upregulation; Parkin-KO no β-cell phenotype	MAMs as mitophagosome formation sites	Whether MAMs control β-cell mitophagy	[43,132,134]

ATP: adenosine triphosphate; Ca^2+^: calcium; dKO: double knockout; eIF2α: eukaryotic translation initiation factor 2α; ER: endoplasmic reticulum; GRP75: glucose-regulated protein 75; GSIS: glucose-stimulated insulin secretion; GSK3β: glycogen synthase kinase 3β; InsP3R/IP3R: inositol 1,4,5-trisphosphate receptor; IRE1α: inositol-requiring enzyme 1α; MAM: mitochondria-associated ER membrane; MCU: mitochondrial calcium uniporter; MERC: mitochondria–ER contact; Mfn2: mitofusin 2; PERK: protein kinase R-like ER kinase; PLA: proximity ligation assay; T2D: type 2 diabetes; UPR: unfolded protein response; VDAC: voltage-dependent anion channel.

## 4. Dysregulated ER–Mitochondria Interactions in Cellular Stresses in β-Cells

The adaptive ER–mitochondria interactions described above can become maladaptive under sustained metabolic stress. The following sections examine how dysregulated crosstalk drives pathological ROS generation and perturbs autophagy and mitophagy in β-cells.

### 4.1. Role of ER-MAM Communication in Generating Reactive Oxygen Species (ROS) in β-Cells

ER–mitochondrial coupling is essential for cellular homeostasis, and its dysregulation contributes to pathological ROS accumulation [135,136,137]. While mitochondrial oxidative phosphorylation normally generates ROS as a signaling molecule, excessive or misregulated ROS production can cause detrimental downstream effects [135,136,137]. MAMs coordinate ER–mitochondrial communication, particularly in Ca^2+^ transfer, to regulate mitochondrial metabolism, redox balance, and apoptosis [138]. Under physiological conditions, this signaling supports controlled ROS turnover; however, excessive or dysregulated ER–mitochondrial calcium flux promotes mitochondrial ROS accumulation, triggering apoptosis and progressive loss of β-cell mass [135,138]. The ER chaperone, Sigma-1 receptor (Sig-1R), is concentrated at the MAMs, and plays a key role in stress sensing and stabilization of Ca^2+^ fluctuations at these contact sites [135].

ER redox enzymes further contribute to the generation of ROS under stress. Endoplasmic reticulum oxidoreductin-1 (ERO1) mediates electron transfer from protein disulfide isomerase (PDI) to a terminal acceptor (molecular oxygen) in the ER pathway, generating H_2_O_2_ as a by-product [139,140]. Although ERO1 expression is upregulated by the UPR to restore ER function, sustained stress leads to excessive ERO1 activity and pathological accumulation of oxidative species [139,140]. In parallel, the adaptor protein p66Shc contributes to oxidative damage by promoting mitochondrial ROS generation and apoptosis. Its activity increases in hyperglycemic states (such as in peripheral blood mononuclear cells from T2D patients, as well as the serum and placenta from women with gestational diabetes) and is regulated by ROS and insulin via serine phosphorylation [141,142]. Deletion of p66Shc showed resistance to oxidative stress-induced apoptosis, which highlights its pathological relevance in diabetes [141,142,143]. Together, these mechanisms reinforce a feed-forward loop in which ER stress enhances ER–mitochondrial coupling and ROS generation, while mitochondrial ROS further exacerbates ER stress, driving β-cell dysfunction and loss [130]. It should be emphasized that this p66Shc evidence derives largely from non-β-cell systems (including peripheral blood mononuclear cells, and serum and placental samples from women with gestational diabetes) rather than from direct β-cell studies; its relevance to the β-cell is therefore inferred by analogy and remains to be established directly.

### 4.2. The Effect of ER–Mitochondria Contact on Autophagy

ER–mitochondria communication can mediate autophagy. Autophagy begins at MERC sites that are important for double-membrane-bound autophagosome formation [144,145]. Hamasaki et al. reported that autophagosomes form at MERC sites in mammalian cells by observing time lapse images in HeLa cells [144]. They found that the pre-autophagosome/autophagosome marker Autophagy-Related 14 (Atg14) re-localized to MERC sites after starvation, and the autophagosome formation marker Atg5 also localized there until autophagosomes had been formed [144]. The ER-resident SNARE protein syntaxin 17 (Stx17 in mammals; Syx17 in flies) acts upstream of Atg14 and recruits Atg14 to the MERC sites forming autophagosomes [144]. Although Atg14 is not yet isolated in pancreatic β-cells, the presence of Atg5 and Atg7 alongside Beclin-1 indicates that a similar complex of autophagosome interacts with the MERC sites in these cells. Autophagosome induction is dependent on ER–mitochondria connections; disrupting these connections by depletion in Mfn2 severely impaired starvation-induced autophagy [146]. A sphingolipid, ganglioside (GD3), is localized to MERC sites [147] and is also involved in initiation of autophagy by binding to the phosphatidylinositol-3-kinase (PI3K) complex and interacting with Microtubule-associated protein 1A/1B-light chain 3 (LC3) after autophagy initiation [148]. Knocking down GD3 synthase led to inhibition in formation of autophagosomes [148]. Thus, autophagic elements expressed in MERC sites are involved in initiation of autophagy.

Associations between ER and mitochondria are regulated by a tethering complex, known as the vesicle-associated membrane protein-associated protein B-protein tyrosine phosphatase-interacting protein 51 (VAPB-PTPIP51) [149]. An ER protein enriched in MAM, vesicle-associated membrane protein-associated protein B (VAPB), binds to the outer mitochondrial membrane protein, protein tyrosine phosphatase-interacting protein 51 (PTPIP51). Gomez-Suaga et al. found that this tethering complex regulates autophagy [150]. Changing VAPB and/or PTPIP51 expression to increase and decrease ER–mitochondria contact affected autophagosome formation; overexpression of VAPB or PTPIP51 to increase ER–mitochondria contact diminished autophagosome formation whereas loss of VAPB or PTPIP51 to decrease contact stimulated autophagosome formation. This implies that their effects on autophagy rely on ER–mitochondria tethering. VAPB-PTPIP51 is involved in Ca^2+^ transport from ER to mitochondria, with several studies showing impairment in IP3R function and Ca^2+^ uptake by mitochondria stimulates autophagy [151,152,153,154]. The function of tethering and Ca^2+^ transport is important to β-cell stress survival, determining whether autophagy perpetuates or halts. The IP3R1-GRP75-VDAC1 tethering complex, whose disruption leads to insulin resistance and autophagy, was characterized in hepatocytes and skeletal muscle rather than in β-cells [155]. Similarly, Gomez-Suaga et al. found that overexpression of VAPB or PTPIP51 increased mitochondrial Ca^2+^ uptake while loss of VAPB or PTPIP51 decreased Ca^2+^ uptake [150], thus revealing another mechanism by which autophagy can be regulated by ER–mitochondria communication. Bosc et al. showed that autophagy occurs at MERC sites to maintain energy metabolism by supplying free fatty acids to mitochondria, while mitochondrial respiratory chain activity supports autophagy through the regulation of MERC formation [156].

Certain MAM proteins can regulate autophagy directly by interacting with key elements of the autophagy mechanism. Mitoguardin (Miga), a mitochondrial outer-membrane protein that regulates MERCs, was recently determined to be essential for autophagy [157]. Miga depletion resulted in accumulation of autophagy markers and substrates along with defects in autophagosome–lysosome fusion whereas Miga overexpression led to Atg14 and Uvrag recruitment to mitochondria. Miga formed complexes by binding to Atg14 and Uvrag to regulate PI3P production and to stabilize Syx17, respectively. These two components are important for regulation of autophagy. Phosphatidylinositol (PI) is the initial substrate required for the PI3K complex to produce PI3P. PI is synthesized on the ER and is transported to the mitochondria via MERC sites [158]. PI3P is involved in autophagosome assembly and activity and thus PI3K promotes formation of PI3P during autophagy [159]. Miga recruits Atg14 to increase PI3P production, a key component of the PI3K complex [157]. Conversely, Syx17 is involved in autophagosome maturation [160], suggesting that Miga stabilizes Syx17 through Atg14 to mediate contact between ER, mitochondria and autophagosomes (Figure 5). How Miga interacts with Atg14 and Atg14 interacts with Syx17, and the exact sites where Miga binds, have not been fully elucidated. Atg14 recruitment, Syx17 interactions have been seen to be important components of the autophagy pathway of pancreatic β-cells, in the presence of knockout mice and mice studies. Beclin-1, for example, is a protein that can recruit the Atg14 presence, which leads to PI3P increase in mice as well [40]. Although Miga itself is not expressed, several proteins of similar nature exist to perform in similar ways; MFN2 and FUNDC1 are proteins expressed in pancreatic β-cells that demonstrate similar results [40]. A small GTPase (Rab32), a key component of MERCs, can also activate autophagy (Figure 5). In 2009, Hirota et al. found that Rab32 facilitates formation of autophagic vacuoles under basal conditions [161]. Interestingly, in 2021, Herrera-Cruz et al. found that Rab32 degrades a subset of MERC-localized proteins through autophagy with the help of reticulon-3 (RTN3L), suggesting its role in selective autophagy of MERCs [162]. These studies indicate the role of MERC components in regulating autophagy.

Disruption in Ca^2+^ transport between ER and mitochondria can also result in increased autophagy. Upon inhibition of ER–mitochondrial Ca^2+^ transfer, AMPK, ADP, AMP and glucose consumption increase while ATP production decreases [163]. In cancer cells, this interruption in Ca^2+^ transfer can result in localized autophagy activation by AMPK via increased Beclin-1 expression [163]. The presence of Beclin-1 and ATG7 in pancreatic β-cells indicates a similar nature of autophagy as described [35]. Another study investigated SEC24A, a protein that facilitates Ca^2+^ transfer between ER and mitochondria and found that inhibiting SEC24A resulted in ~2.5-fold increase in autophagic flux [164]. In pancreatic β-cells, it has been seen that the etoposide-induced gene 2.4 protein (EI24) is found in MAMs, interacting with IP3 and leading to autophagy flux in pancreatic β-cells [164]. Moreover, the depletion of a subunit of a protein complex TOM, the mitochondrial translocase of the outer membrane 70 (TOM70), impaired ER–mitochondria Ca^2+^ transfer and induced autophagy [165]. Thus, MERC sites serve as an induction site for autophagosomes, and disruption in ER–mitochondria communication can result in autophagy.

### 4.3. The Effect of ER–Mitochondria Contact on Mitophagy

MERCs are also closely related to mitophagy regulation and induction. A complex of Mmm1–Mdm12, two core subunits of the ER–mitochondrial encounter structure (ERMES) in yeast, was reported to be essential for phospholipid transfer between membranes in vitro [166]. Ubiquitination of Mdm34 and Mdm12 are required for efficient mitophagy, implying that ER–mitochondria coupling is involved in mitophagosome formation. The importance of MERCs in relation to mitophagy is seen in β-cells. ATF4 regulates the expression of Parkin, a ubiquitin ligase that carries out mitophagy during ER stress [167]. Parkin is also involved in the ER–mitochondria interaction during ER stress since it enhances MAM microdomain assembly for maintaining intra-organelle Ca^2+^ transfer [168]. This suggests a role of MAM proteins in the induction of mitophagy in β-cells. Moreover, Yeshaw et al. investigated vacuolar protein sorting (VPS) 13A, which is localized at MERC sites and found that depletion of VPS13A resulted in reduced MAM formation and fragmented mitochondria, along with decreased mitophagy [169], suggesting that MERC sites play a role in mitophagy.

Components of the MERC sites can also regulate mitophagy. For example, TraB family proteins are found at MERCs and can, in fact, regulate ER–mitochondrial interaction [170]. Interestingly, they can also interact with Atg8 and act as mitophagy receptors by regulating mitochondrial degradation [170]. ATF4 can enhance autophagy activity by promoting Parkin expression [171]; Parkin can ubiquitinate mitochondrial membrane proteins through E3 ubiquitin ligase activity to recruit p62 protein, which subsequently interacts with LC3 to cause mitophagy [172]. The SNARE protein Stx17 (found at MERC sites) is involved in autophagosome formation and initiates mitophagy upon depletion of outer mitochondrial membrane protein Fis1 [173]. The presence of ATF4 and Parkin in pancreatic β-cells is suggestive of pathways of mitophagy present in these cells; ER stress leads to activation of ATF4 and Parkin in a similar pathway, leading to mitophagy if uncontrolled or dysregulated [174] (Figure 5).

Interestingly, reducing ER–mitochondria contact can increase the rate of mitophagy. Components involved in the induction of mitophagy uncouple ER and mitochondria. McLelland et al. revealed this Parkin-/PINK1-dependent mechanism, which regulates the destruction of MERC sites [175]. Parkin/PINK1 caused MFN2 to be phospho-ubiquitinated during autophagy [175]. This initiates the dismantling of Mfn2 complexes from the outer mitochondrial membrane via p97, leading to separation of mitochondria from ER. This implies a possibility of cell death due to deletion of PINK1 following increased mitochondrial Ca^2+^ sensitivity, thus implying a need to adjust the mitochondrial content of PINK1 to regulate Parkin-dependent mitophagy [175]. Another study on human umbilical vein endothelial cells (HUVECs) validated these results, finding that ER stress led to mitochondrial Ca^2+^ overload and accumulation of Parkin and p62 in mitochondria [176] that stimulated mitophagy. Obese patients with newly diagnosed type 2 diabetes exhibit significantly reduced PINK1 gene expression paired with a compensatory elevation in serum total antioxidant response compared to lean patients [177]. In mice, PINK1 deficiency impairs pancreatic β-cell function by elevating basal calcium levels and inhibiting glucose transport, which drives glucose-independent hypersecretion of basal insulin while leaving the downstream mitochondrial pathway to insulin secretion intact [178]. Overall, these studies suggest that uncoupling of ER and mitochondria is necessary for efficient mitophagy and highlights the role of MERCs in this process. Table 3 summarizes studies elucidating the role of ER–mitochondria associations in autophagy and mitophagy.

## 5. Comparative Analysis of ER–Mitochondria Interaction in Human and Mouse β-Cells

### 5.1. Comparative Models Used to Study MAMs in β-Cells

Most mechanistic work on ER–mitochondria contact sites in β-cells derives from rodent models or established cell lines: the MIN6 mouse line and INS-1E rat line in particular. The MIN6 line, derived from a mouse insulinoma, retains glucose-responsive insulin secretion and is widely used to dissect β-cell signaling, whereas the INS-1E clone, selected from rat INS-1 insulinoma cells for its stable and robust glucose-stimulated insulin secretion, is a preferred rodent model for metabolic and mitochondrial studies. Neither line fully reproduces the transcriptional profile, ploidy or Ca^2+^ handling of primary human β-cells [181], which constrains their translational reach. Whether these findings translate to human β-cells is not guaranteed, and data from human islets sometimes diverges from rodent predictions. Thivolet et al. examined ER–mitochondria interactions in β-cells from nPOD donors (12 individuals with T2D and nine non-diabetic controls) and found IP3R2 protein and mRNA significantly elevated in T2D β-cells, while VDAC1 expression was substantially reduced [55]. The number of functional IP3R2-VDAC1 complexes, assessed by proximity ligation assay, was significantly lower in T2D islets. This contrasts with MIN6 cells under palmitate, which showed increased ER stress but a distinct protein expression pattern, suggesting that cellular context and the mode of metabolic challenge both matter. Dingreville et al. showed that while chronic high-glucose exposure reduced Ca^2+^ exchange at ER–mitochondria contact sites in both INS-1E cells and human islets, the kinetics and magnitude of the Ca^2+^ response differed between the two systems [54]. These species differences carry direct translational consequences. Human islets contain roughly 60% β-cells compared to ~80% in mouse islets, and their cytoarchitecture, with alpha cells distributed throughout rather than at the periphery, creates a different local signaling environment that likely affects MAM site regulation [182]. Transcriptomic studies have documented substantial differences between human and mouse β-cells in genes encoding MAM tethering and regulatory proteins [181,183]. A central unresolved question is whether the MAM disruptions observed in diabetic mouse models, including altered PDZD8 expression and IP3R1-GRP75-VDAC1 complex stoichiometry, are replicated in human T2D islets at a functionally equivalent level.

### 5.2. Comparative MAM Composition and Glucotoxic Response

Although no proteins are exclusively localized at MAMs, the relative abundance of tethering components (IP3R, VDAC1, GRP75, MFN2, and PACS-2) shapes organelle coupling in a species- and stress-dependent manner [184]. Comparative proteomic profiling of MAMs in a long-term diabetic rodent model identified 1239 quantifiable proteins, of which 144 were significantly altered in diabetes, mapping to Ca^2+^ signaling, ER stress, inflammation, and metabolism pathway clusters [185]. Notably, the functional consequences of MAM dysregulation diverge between species. In INS-1E cells and murine islets, acute glucose stimulation enhances MAM assembly and ATP-stimulated Ca^2+^ exchange, supporting GSIS [21,54]. However, chronic high glucose paradoxically increases structural organelle tethering while decreasing functional Ca^2+^ transfer, accompanied by ER stress; altered mitochondrial respiration; and mitochondrial fission [186]. In human β-cells, chronic glucose stress induces markedly different early responses: augmented basal mitochondrial respiration, contrasting with reduced respiration in islets from diabetic donors [187].

### 5.3. Morphological Correlates

Ultrastructural studies further emphasize species differences. Human T2D β-cells display round-shaped mitochondria with altered cristae, increased volume, and reduced electron density, along with expanded ER density compared with non-diabetic controls [188]. Quantitative serial block-face scanning electron microscopy of mouse islets has shown that β-cell ER cisternae are less prominent than those of α-cells and extend only ~0.8 μm in cross-section, providing the structural context within which MAM contacts operate [189]. Table 4 compares key ER–mitochondria parameters between human β-cells and mouse models or cell lines across the major topics covered in this review.

## 6. Therapeutic Approaches Targeting ER and Mitochondria

Incretin-based therapies, particularly glucagon-like peptide-1 (GLP-1) receptor agonists, are increasingly used in the treatment of T2D due to their ability to enhance glucose-dependent insulin secretion, suppress glucagon release and improve β-cell survival [191,192,193,194,195,196]. Beyond their classical effects on islet hormone secretion, emerging evidence suggests that GLP-1 signaling directly modulates cellular metabolism through regulation of ER–mitochondria crosstalk. Specifically, GLP-1 has been shown to enhance ER–mitochondria coupling via protein kinase A (PKA)-dependent upregulation of Mfn2, leading to increased mitochondrial Ca^2+^ uptake, oxidative metabolism, ATP production and oxygen consumption [196]. The role of Mfn2 at contact sites is the clearest example: initial work proposed that Mfn2 physically tethers ER and mitochondria such that its ablation reduces contact frequency [197], while subsequent data showed Mfn2 ablation actually increased ER–mitochondria proximity, implying Mfn2 acts as a spacer rather than a bridge [89]. Estrogen receptor α (ERα) may represent an additional therapeutic node, particularly relevant for women with T2D. In ERα-knockout mouse islets and ERα-silenced MIN6 cells, loss of receptor function impaired mitochondrial fission/fusion dynamics, increased ROS, and disrupted ER morphology, a pattern consistent with defective MAM organization [51]. ERα overexpression or ligand activation reduced ER stress markers and protected against lipotoxicity-induced apoptosis. These data identify a link between sex hormone signaling and organelle crosstalk in β-cells, with potential implications for sex-specific differences in T2D progression and susceptibility. Partial PERK inhibition improved glucose-stimulated insulin secretion in both mouse and human islets by enhancing cytosolic Ca^2+^ transit [97], suggesting PERK is a calibratable target rather than a simple on/off switch. Reducing PDZD8-mediated ER–mitochondria tethering lessened organelle stress in experimental diabetic conditions [60], indicating that excessive MAM contact, not only insufficient contact, can be pathological. Ageing warrants brief but explicit consideration, since age is the dominant risk factor for T2D and two cardinal hallmarks of ageing (mitochondrial dysfunction and loss of proteostasis) are precisely the processes that remodel the ER–mitochondria interface [184]. β-cells accumulate senescence markers with age and insulin resistance, and genetic or pharmacological clearance of senescent β-cells (senolysis) improves glucose tolerance and insulin secretion, establishing β-cell senescence as a driver, not merely a correlate, of diabetes [198]. Mitochondrial-derived peptides may couple this ageing program to organelle crosstalk: MOTS-c declines with age and senescence in islet cells and in the circulation of patients with T2D, and MOTS-c treatment reduces islet senescence and improves glucose tolerance in aged and diabetic mice, plausibly acting through MAM-associated Ca^2+^ and redox pathways [190]. These observations justify treating ER–mitochondria coupling as a candidate determinant of β-cell longevity while acknowledging that direct evidence linking MAM remodeling to β-cell ageing is still limited. Table 5 distinguishes these approaches according to whether they represent potential therapeutic targets or strategies already supported by therapeutic evidence, and specifies the level of evidence (cellular, animal, or patient) in each case.

## 7. Conclusions and Future Perspectives

Mitochondrial dysfunction and ER stress have been implicated in the pathogenesis of diabetes, and in particular β-cell failure, for decades. The mitochondria–ER relationship has emerged as a novel avenue for investigation in the pathogenesis of T2D. β-cells are limited in their protection capacity against high levels of stress, undergoing functional and morphological changes under stressful conditions. ER–mitochondria communication affects β-cell stress and dysfunction, especially through the expression of MAM proteins since these are associated with furthering the stress-induced damage to β-cells and are responsible for insulin signaling and action. However, the exact effect of MERCs on β-cell stressors in human β-cells awaits direct investigation. Nevertheless, the concept of mitochondria–ER coupling is a novel area for future investigation, from both a biological and therapeutic perspective. Each protein and gene involved in β-cell stress is of interest for future pharmacological development, especially proteins involved with the mitochondria of β-cells. Future investigations into the modulation of MAM proteins and MAM alterations under hyperglycemic stimulation may provide novel therapeutic treatments for diabetes.

## Figures and Tables

**Figure 1:**
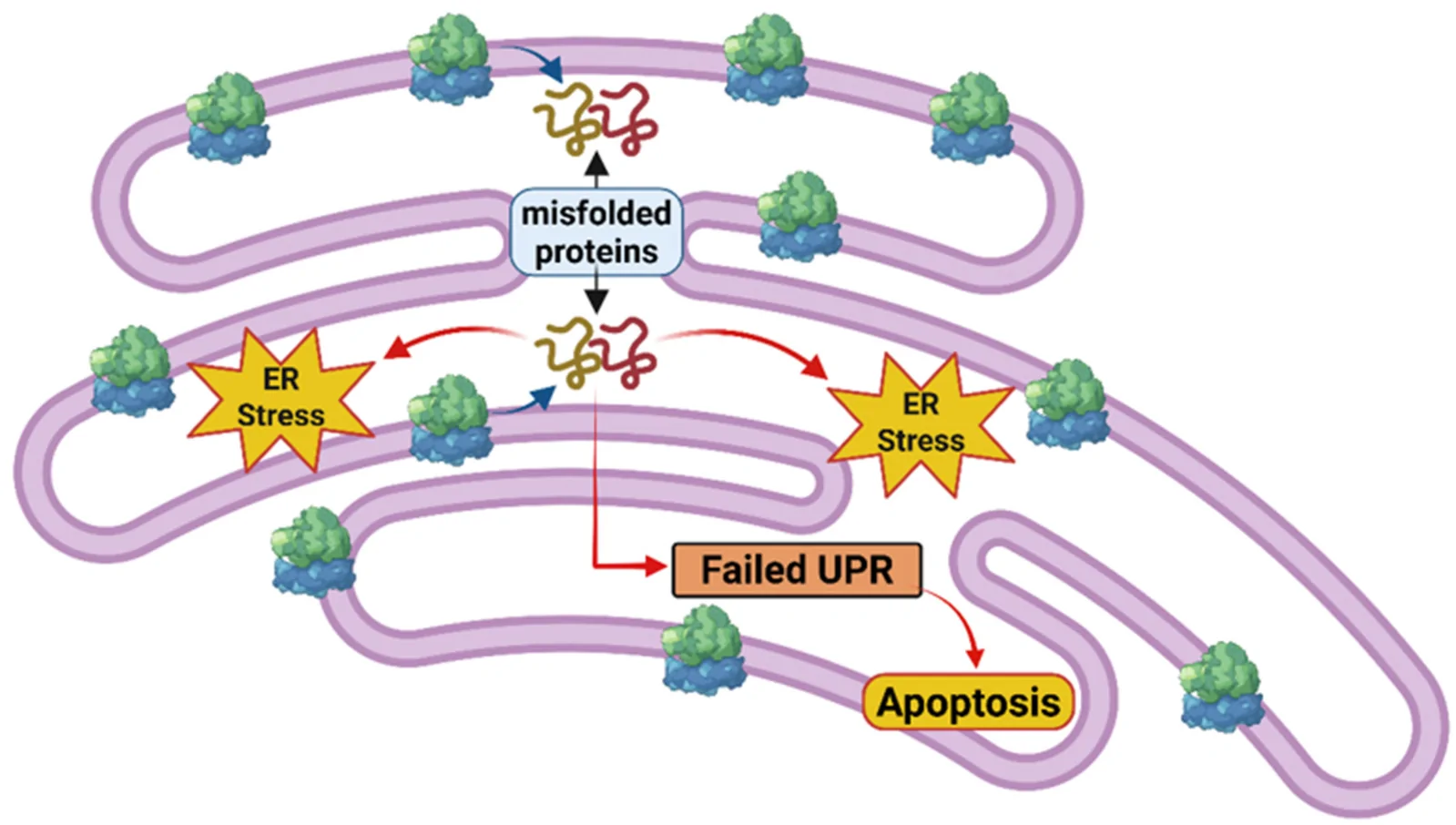
The overwhelmed UPR in the ER. UPR works to clear misfolded proteins in the ER and maintain cellular homeostasis [6]. Under chronic or excessive stress, however, the UPR loses its protective capacity and triggers apoptosis, mirroring the response of β-cells in T2D [7,23,24,25]. This figure was created with BioRender.com. (UPR: unfolded protein response; ER: endoplasmic reticulum; T2D: type 2 diabetes). In brief, the schematic contrasts the protective, homeostatic UPR (which restores ER proteostasis) with the maladaptive UPR that predominates under chronic stress and drives β-cell apoptosis in T2D. Different arrow colors are used strictly to improve visual distinction, not to signify different categories or values.

**Figure 2:**
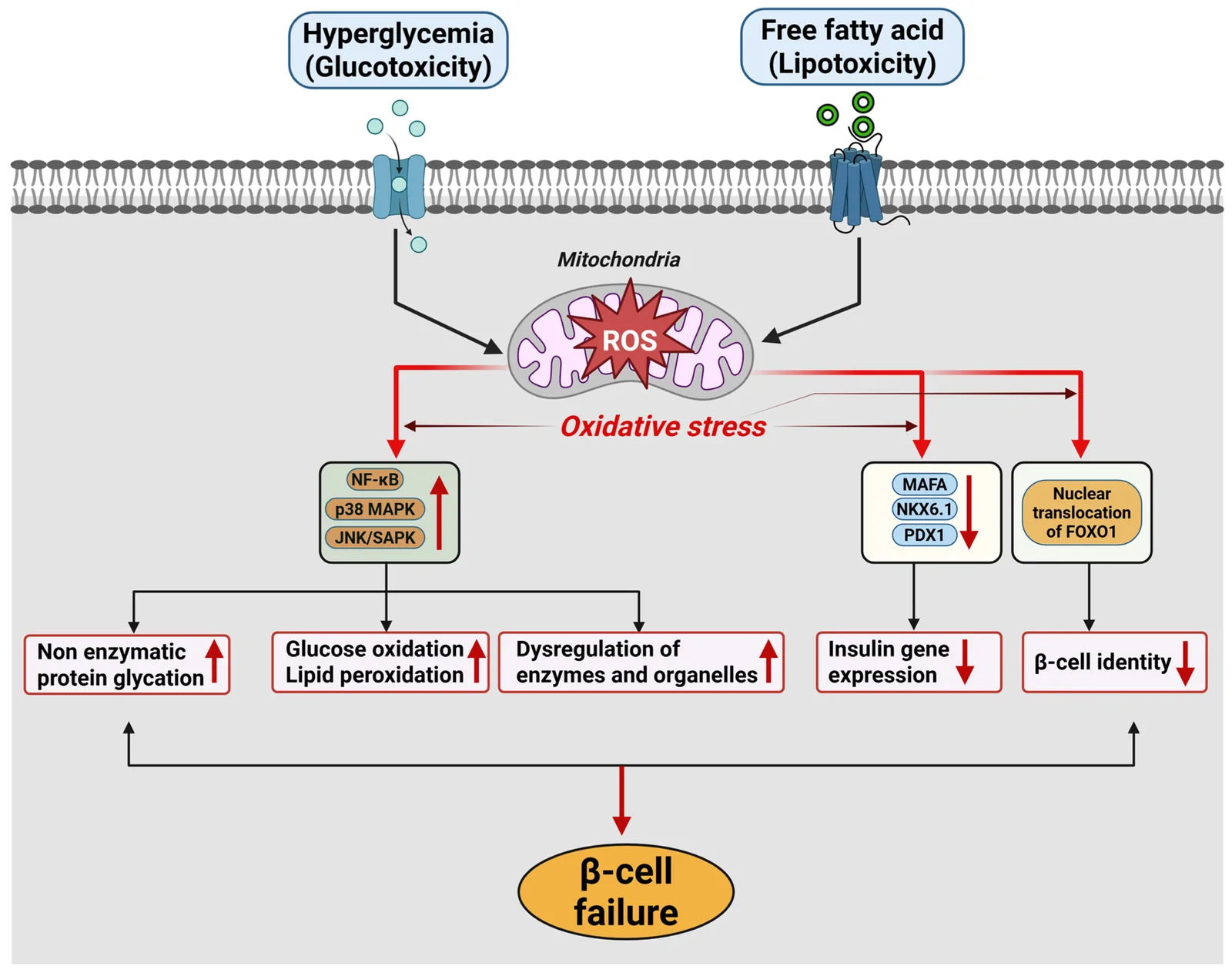
Pathological pathway leading to β-cell failure in T2D. In T2D, excessive glucose and lipid accumulation in pancreatic β-cells leads to elevated ROS production and activation [26,27,28]. Oxidative stress activates three major stress pathways: nuclear factor-κB (NF-κB), p38 mitogen-activated protein kinase (p38 MAPK) and NH_2_-terminal Jun kinases/stress-activated protein kinases (JNK/SAPK). With persistent activation and upregulation of these pathways, free radicals are increased by non-enzymatic protein glycation, glucose oxidation and lipid peroxidation. These free radicals continue to dysregulate enzymes and organelles within the β-cell, further leading to β-cell impairment and loss [26,27,28]. In parallel, oxidative stress lowers the β-cell identity transcription factors MAFA, NKX6.1 and PDX1 and drives nuclear translocation of FOXO1, reducing insulin gene expression and β-cell identity. This figure was created with BioRender.com. (T2D: type 2 diabetes; NF-κB: nuclear factor-κB; p38 MAPK: p38 mitogen-activated protein kinase; JNK/SAPK: NH_2_-terminal Jun kinases/stress-activated protein kinases; ROS: reactive oxygen species; MAFA: MAF bZIP transcription factor A; NKX6.1: NK6 homeobox 1; PDX1: pancreatic and duodenal homeobox-1; FOXO1: forkhead box O1). Different arrow colors are used strictly to improve visual distinction, not to signify different categories or values.

**Figure 3:**
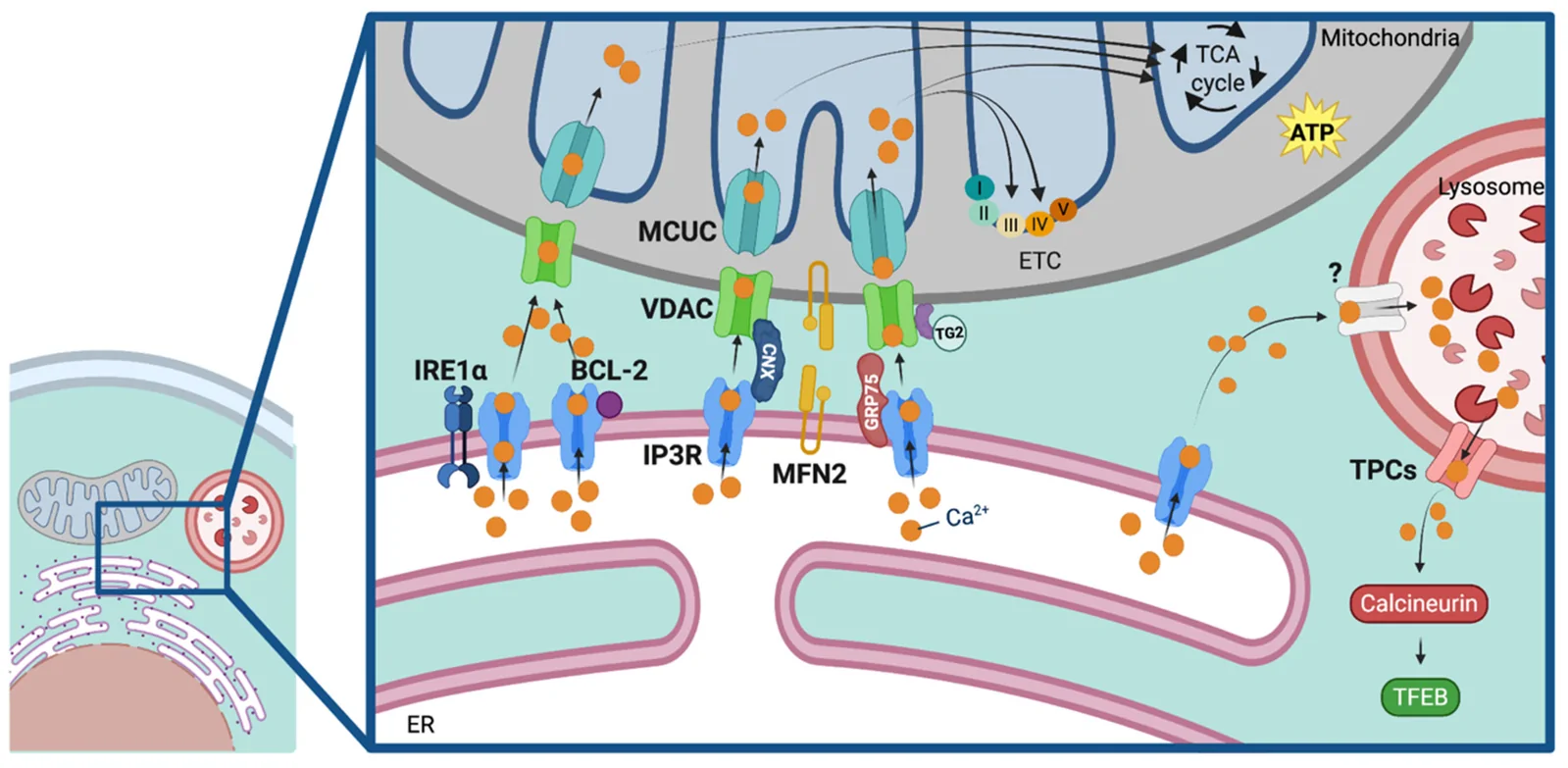
Calcium signaling at ER–mitochondria contact site. The endoplasmic reticulum (ER) serves as the primary storage site for cellular Ca^2+^, which is subsequently transferred to mitochondria. This transfer is facilitated by inositol 1,4,5-trisphosphate receptor (IP3R) channels, which move Ca^2+^ from the ER lumen to mitochondria through interaction with the mitochondrial voltage-dependent anion channel 1 (VDAC1) on the outer mitochondrial membrane (OMM). IP3R interacts with proteins such as IRE1α, BCL-2 family proteins, calnexin (CNX) and Chaperone glucose-regulated protein 75 (Grp75), which mediate in the regulation of Ca^2+^ transfer [87]. Ca^2+^ then enters the inner mitochondrial membrane (IMM) via the low-affinity mitochondrial Ca^2+^ uniporter complex (MCUC) and regulates the tricarboxylic acid (TCA) cycle and electron transport chain (ETC) complexes [88]. The mitochondrial fusion protein Mitofusin2 (MFN2), located on both the ER and the OMM, also plays a pivotal role in regulating the proximity of ER–mitochondria contact sites and the flux of Ca^2+^ between these organelles [89,90]. Additionally, IP3R-mediated Ca^2+^ release may pass through the lysosomal membrane through an unidentified channel (?), which releases lysosomal Ca^2+^ that regulates calcineurin activity, leading to downstream events such as the nuclear translocation of Transcription factor EB (TFEB) [91]. This figure was created with BioRender.com. (ER: endoplasmic reticulum; IP3R: inositol 1,4,5-trisphosphate receptor; VDAC: voltage-dependent anion channel; OMM: outer mitochondrial membrane; Grp75: Chaperone glucose-regulated protein 75; IMM: inner mitochondrial membrane; MCUC: mitochondrial calcium uniporter complex; TCA: tricarboxylic acid cycle; ETC: electron transport chain; MFN2: mitofusin 2; TFEB: Transcription factor EB; BCL-2: B-cell lymphoma 2; CNX: calnexin; IRE1α: inositol-requiring enzyme 1α; VDAC1: voltage-dependent anion channel 1; TG2: transglutaminase 2; TPCs: two-pore channels). Different symbol colors are used strictly to improve visual distinction, not to signify different cat-egories or values.

**Figure 4:**
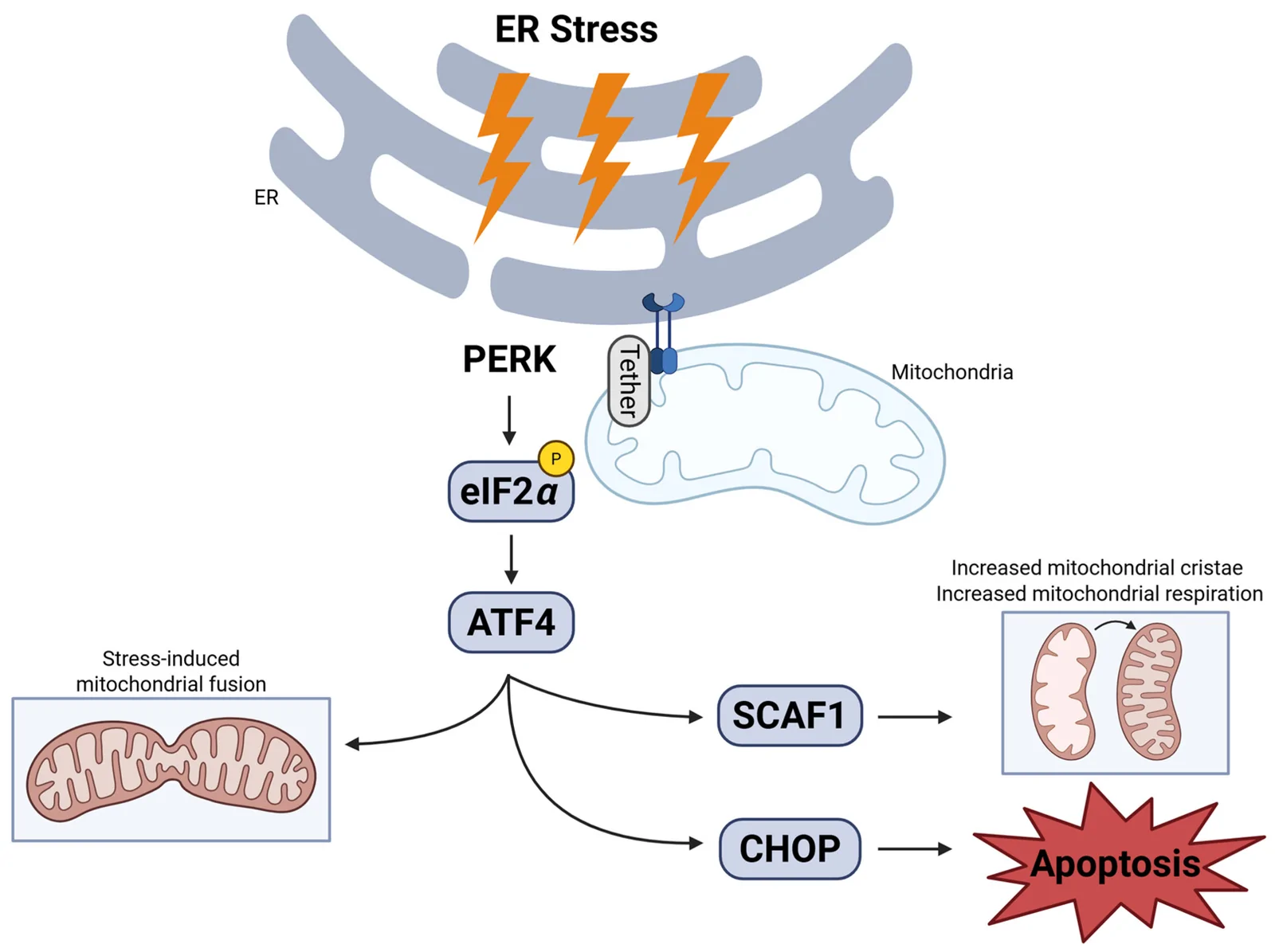
PERK signaling at ER–mitochondria contact sites. PERK takes on several roles in ER stress signaling pathways. PERK is required to tether the ER to the mitochondrial membrane at the MAMs, aiding the transfer of ROS signaling [118]. Additionally, upon exposure to ER stress, PERK phosphorylates eukaryotic initiation factor 2 alpha (eIF2*α*), allowing for the expression of activating transcription factor 4 (ATF4), which leads to C/EBP homologous protein (CHOP) induction and apoptosis. The PERK-eIF2α-ATF4 axis can also increase supercomplex assembly factor 1 (SCAF1), which leads to the formation of respiratory supercomplexes, increasing the mitochondrial cristae and thus increasing mitochondrial respiration [119]. ER stress also triggers protective mitochondrial elongation in the form of mitochondrial fusion. This figure was created with BioRender.com. (PERK: protein kinase receptor-like endoplasmic reticulum kinase; MAMs: mitochondria-associated ER membranes; ER: endoplasmic reticulum; ROS: reactive oxygen species; eIF2*α*: eukaryotic initiation factor 2 alpha; ATF4: activating transcription factor 4; CHOP: C/EBP homologous protein; SCAF1: supercomplex assembly factor 1). Different symbol colors are used strictly to improve visual distinction, not to signify different cat-egories or values.

**Figure 5:**
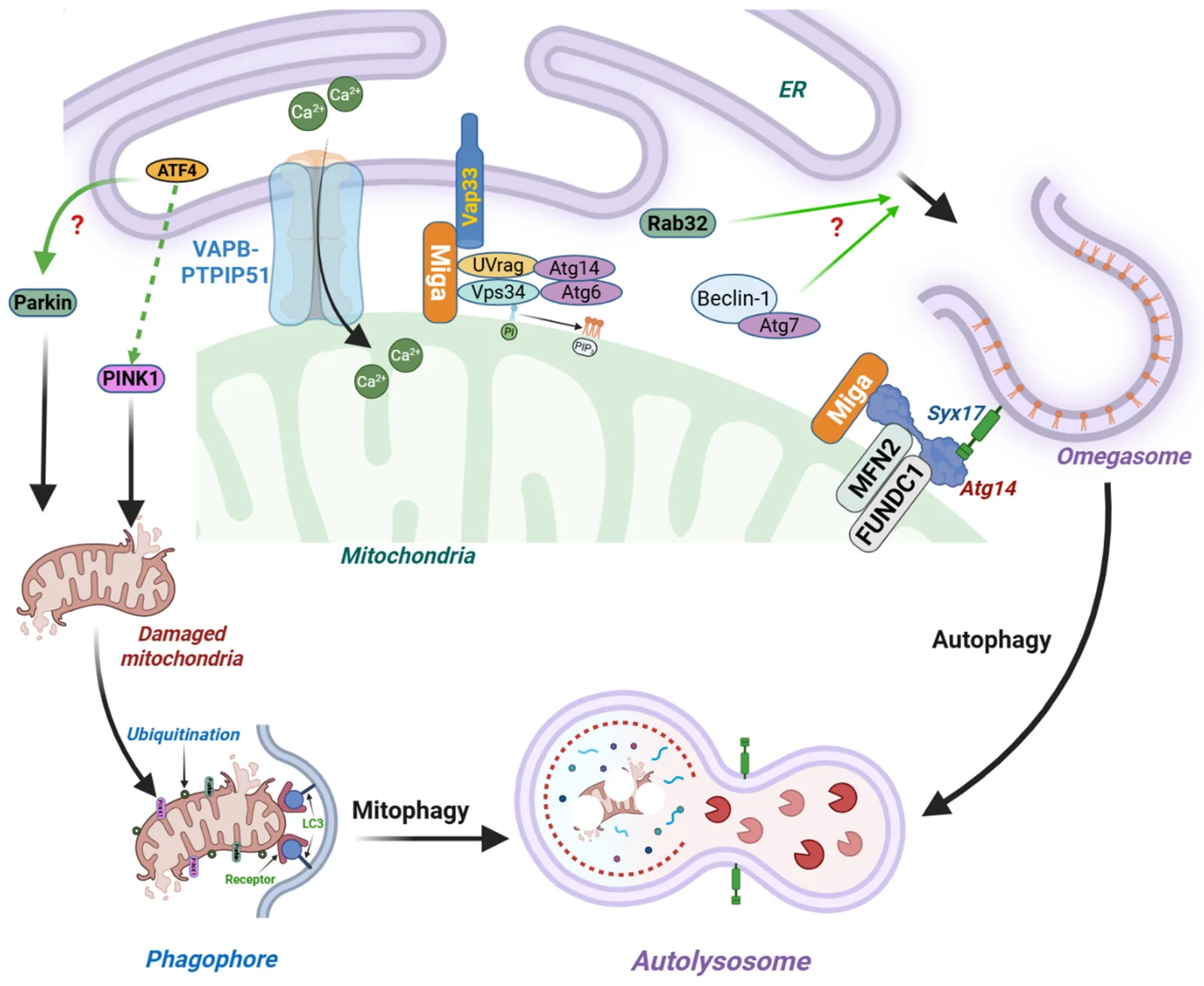
ER–mitochondria contact sites (MERCs) coordinate autophagy and mitophagy in pancreatic β-cells. The endoplasmic reticulum (ER) and mitochondria are physically linked at mitochondria-associated membranes (MAMs)/mitochondria–ER contact sites (MERCs), which serve as platforms for both autophagosome formation (right) and the clearance of damaged mitochondria by mitophagy (left). Central to the diagram, the ER and the outer mitochondrial membrane are tethered by the VAPB–PTPIP51 complex (vesicle-associated membrane protein-associated protein B–protein tyrosine phosphatase-interacting protein 51), which mediates Ca^2+^ transfer from the ER to the mitochondria. *Right side*, Autophagy. Mitoguardin (Miga), a mitochondrial outer-membrane protein that regulates MERCs, binds Vap33 (VAMP-associated protein 33) and recruits UVrag (UV radiation resistance-associated gene protein) together with the class III phosphatidylinositol-3-kinase (PI3K) complex components Vps34 (vacuolar protein sorting 34), Atg6/Beclin-1 (Autophagy-Related 6), and Atg14 (Autophagy-Related 14). This complex converts phosphatidylinositol (PI) to phosphatidylinositol-3-phosphate (PIP_3_/PI3P), which drives autophagosome assembly. A separate pool of Miga associates with MFN2 (Mitofusin 2) and FUNDC1 (FUN14 domain-containing protein 1) to stabilize Syx17/Stx17 (syntaxin 17) and Atg14 (Autophagy-Related 14) at the contact site, mediating contact between the ER, mitochondria, and forming autophagosomes. The small GTPase Rab32 (Ras-related protein Rab-32) and the Beclin-1 (Coiled-coil myosin-like BCL2-interacting protein)–Atg7 (Autophagy-Related 7) complex further promote autophagy initiation; question marks (?) denote proposed or incompletely defined steps in β-cells. These events nucleate the omegasome and the double-membrane phagophore that matures into the autophagosome and ultimately fuses with the lysosome to form the autolysosome, where cargo is degraded (Autophagy). *Left side*, Mitophagy. Under ER stress, the transcription factor ATF4 (activating transcription factor 4) induces expression of Parkin (E3 ubiquitin–protein ligase parkin) and, together with PINK1 (PTEN-induced putative kinase 1), targets damaged mitochondria for degradation; the green dashed and solid arrows with question marks (?) indicate proposed regulatory links. On the damaged mitochondria, PINK1 (PTEN-induced putative kinase 1) and Parkin (E3 ubiquitin–protein ligase parkin) drive ubiquitination of outer mitochondrial membrane proteins, recruiting the autophagy receptor p62/SQSTM1 (sequestosome 1), which engages LC3 (Microtubule-associated protein 1A/1B-light chain 3) on the encircling phagophore. The engulfed mitochondrion is delivered to the autolysosome for degradation (Mitophagy). Question marks in the illustration indicate that the pathways are suggestive in pancreatic β-cells and yet to be confirmed. Different arrow and symbol colors are used strictly to improve visual distinction, not to signify different cat-egories or values.

**Table 1:** Main molecular regulators of ER–mitochondria contacts (MAMs/MERCs) discussed in this review. The column “Evidence in β-cells” cites the manuscript reference in which direct β-cell evidence is available; “Not available” would indicate that, to our knowledge, no direct β-cell evidence exists, and the role is inferred from other cell types.

Regulator	Localization	Proposed Function	Effect on Ca^2+^	Experimental Model(s)	Evidence in β-Cells
IP3R (IP3R1/IP3R2)	ER membrane (MAM)	ER Ca^2+^-release channel; core of the IP3R–GRP75–VDAC1 transfer complex	Releases ER Ca^2+^ for transfer to mitochondria (pro-transfer)	Human islets/nPOD tissue; INS-1E	[21]
VDAC1	Outer mitochondrial membrane	Mitochondrial Ca^2+^-conducting channel; docks to IP3R via GRP75	Conducts ER-derived Ca^2+^ into mitochondria	Human islets/nPOD; mouse β-cells (high glucose)	[21,80]
GRP75	Cytosol/MAM (bridging chaperone)	Molecular bridge physically coupling IP3R to VDAC1	Enables and stabilizes ER→mitochondria Ca^2+^ transfer	Inferred from the IP3R–VDAC1 complex quantified in human islets	[21]
Mfn2	Outer mitochondrial membrane and ER	Tethering/spacing of ER–mitochondria contacts; mitochondrial fusion	Context-dependent regulation of ER–mito Ca^2+^ flux (tether vs. spacer)	Mouse β-cells/islets (mitofusin KO); INS-1	[89,90]
IRE1α	ER membrane (UPR sensor at the MAM)	UPR transducer (XBP1 splicing, RIDD); couples ER stress to contacts	Modulates [Ca^2+^]ER and IP3R-mediated transfer	Mouse β-cells (β-cell-specific deletion); islets	[96]
PERK	ER membrane (UPR sensor, MAM-enriched)	UPR transducer (eIF2α–ATF4); ER–mito tether and ROS relay	Influences ER–mito Ca^2+^/ROS coupling	Mouse and human islets (partial inhibition)	[97,98]
JPH3 (junctophilin-3)	ER/junctional membrane	Structural ER–mitochondria junction protein	Maintains ER–mito spatial organization and Ca^2+^ handling	Mouse and human β-cells (knockdown)	[59]
PDZD8	ER membrane (tether)	ER–mitochondria tethering (SMP domain lipid transfer/tether protein)	Augments mitochondrial Ca^2+^ uptake	β-cells under experimental diabetic conditions	[60]

ATF4: activating transcription factor 4; Ca^2+^: calcium ion; eIF2α: eukaryotic translation initiation factor 2α; ER: endoplasmic reticulum; GRP75: glucose-regulated protein 75; INS-1/INS-1E: rat insulinoma β-cell line (clone 1E); IP3R (IP3R1/IP3R2): inositol 1,4,5-trisphosphate receptor (type 1/2); IRE1α: inositol-requiring enzyme 1α; JPH3: junctophilin-3; KO: knockout; MAM: mitochondria-associated ER membrane; MERC: mitochondria–ER contact site; Mfn2: mitofusin 2; nPOD: Network for Pancreatic Organ Donors with Diabetes; PDZD8: PDZ domain-containing protein 8; PERK: protein kinase R-like ER kinase; RIDD: regulated IRE1-dependent decay; ROS: reactive oxygen species; UPR: unfolded protein response; VDAC1: voltage-dependent anion channel 1; XBP1: X-box binding protein 1.

**Table 3:** Summary of the major studies investigating the association between ER–mitochondria contact sites and autophagy and mitophagy.

Degradation Pathway	Experimental Condition	Findings	Direct ER–Mito Contact Assessed?	References
Autophagosome formation	Fasting–feeding; mTORC1 modulation	mTORC1 dynamically regulates β-cell autophagy across fasting–feeding cycles; loss or chronic hyperactivation of mTORC1 disrupts autophagy and β-cell function.	No (autophagy machinery)	[39]
Islet homeostasis/β-cell mass	High-fat diet; db/db	Compensatory upregulation of β-cell autophagy under high-fat feeding; Atg7 deletion causes islet degeneration and glucose intolerance from lack of compensatory β-cell mass.	No (autophagy machinery)	[38]
β-cell mass and function	β-cell Atg7 knockout	Loss of autophagy reduces β-cell mass and insulin content, impairs glucose-stimulated secretion and Ca^2+^ transients, with ubiquitinated aggregates, mitochondrial swelling and ER distension.	Indirect (ER/mito ultrastructure)	[37]
Autophagosome and mitophagosome formation	ER stress (ATF4/eIF2α axis)	The eIF2α–ATF4 pathway is required for stress-induced autophagy gene expression, coupling ER stress to autophagy/mitophagy induction.	No (autophagy machinery)	[98]
Mitophagosome formation	Glucose challenge	PINK1 knockout reduces NADH generation and mitochondrial potential	No (mitophagy machinery)	[178]
Autophagosome formation	Atg5/Atg7 knockout	Atg5/Atg7 knockout disrupts autophagosome formation, causing β-cell dysfunction and impaired glucose tolerance.	Indirect (organelle morphology)	[40]
Autophagy vs. apoptosis	Beclin-1/IP3R interaction	IP3R regulates autophagy through its interaction with Beclin-1; graded loss of Beclin-1 shifts the balance between autophagy and apoptosis (severe depletion increases cell death).	Yes (IP3R–Beclin-1 at ER)	[151]
Tethering complex	Reduced tether expression	The IP3R–GRP75–VDAC1 complex mediates ER-to-mitochondria Ca^2+^ transfer; its disruption impairs insulin signaling and induces autophagy, shown in hepatocytes and skeletal muscle; in human β-cells the analogous IP3R2–VDAC1 complex is reduced in T2D.	Yes (PLA in β-cells/MAM integrity)	[55,179,180]
Lipotoxic MAM remodeling	Saturated vs. unsaturated FFAs in INS-1E cells	Palmitate and stearate increase ER–mitochondria contacts through H_2_O_2_-dependent MAM remodeling; GPX8 blunts this, and forced ER–mitochondria linking increases lipotoxic cell death.	Yes (PLA, TEM, genetically encoded MAM sensors)	[56]

ATF4: activating transcription factor 4; db/db: leptin receptor-deficient diabetic mouse; eIF2α: eukaryotic initiation factor 2α; ER: endoplasmic reticulum; FFA: free fatty acid; GRP75: glucose-regulated protein 75; IP3R (IP3R1/IP3R2): inositol 1,4,5-trisphosphate receptor (type 1/2); mTORC1: mechanistic target of rapamycin complex 1; NADH: nicotinamide adenine dinucleotide (reduced); PINK1: PTEN-induced kinase 1; PLA: proximity ligation assay; T2D: type 2 diabetes; VDAC1: voltage-dependent anion channel 1; Beclin-1: coiled-coil myosin-like BCL2-interacting protein; GPX8: glutathione peroxidase 8; TEM: transmission electron microscopy.

**Table 4:** Comparative summary of key ER–mitochondria interaction parameters in mouse models/cell lines versus human islets.

Parameter	Mouse Model/Cell Line	Human Islet	Reference
ER–mitochondria contact frequency under glucotoxicity	Increased contacts in Min6-B1 under palmitate; acute glucose stimulates coupling but chronic high glucose reduces Ca^2+^ exchange in INS-1E	Reduced IP3R2-VDAC1 complexes (PLA) in nPOD T2D donors; chronic high glucose reduces Ca^2+^ exchange in human islets	[54,55]
IP3R2 expression in T2D	Palmitate-induced ER stress in Min6-B1; IP3R function in Ca^2+^ signaling established in islet cell lines	Significantly elevated at mRNA and protein level in T2D β-cells (nPOD donors)	[55]
VDAC1 expression in T2D	Reduced under glucolipotoxicity in Min6-B1	Significantly reduced in T2D β-cells (nPOD donors)	[55]
Effect of PERK inhibition on GSIS	Improved glucose-stimulated insulin secretion in mouse islets	Improved glucose-stimulated insulin secretion in human islets	[97]
Mfn2 loss of function	ER stress, UPR activation, reduced ER–mitochondria coupling in mouse islets	Reduced mitochondrial volume density in palmitate-treated human islets (correlated with lower PERK levels)	[100,124]
Estrogen receptor α (ERα)	ERα KO disrupts mitochondrial fission/fusion-mitophagy dynamics in mouse islets and MIN6 cells	Not yet characterized in primary human islets	[51]
ER–mitochondria crosstalk in T1D	Cytokine-induced concurrent ER stress and mitochondrial dysfunction characterized in mouse islet models	Not yet characterized in primary human T1D islets	[21]
Aging and islet MAM biology	MOTS-c declines in aged C57BL/6 mouse islets; MOTS-c treatment reduces islet senescence markers and improves glucose tolerance	Lower circulating MOTS-c in T2D patients vs. healthy controls	[190]
Mitofusin (Mfn1/Mfn2) loss of function	β-cell Mfn1/2 double-knockout causes β-cell loss, fragmented mitochondria, reduced mtDNA and defective glucose- (but not incretin-) stimulated insulin secretion and connectivity.	Not yet characterized in primary human islets	[102]
PDZD8-dependent MAM formation	PDZD8 knockdown reduces MAM perimeter and VDAC1–IP3R1 interactions in diabetic mouse islets and palmitate-treated INS-1 cells, limiting mitochondrial Ca^2+^ overload and β-cell death.	Not yet characterized in primary human islets.	[60]
Lipotoxic MAM remodeling	Saturated FFAs increase ER–mitochondria contacts in INS-1E cells via H_2_O_2_-dependent MAM remodeling; unsaturated FFAs counteract this.	Not yet characterized in primary human islets.	[56]

(Ca^2+^: calcium ion; C57BL/6: C57 black 6 inbred mouse strain; ER: endoplasmic reticulum; ERα: estrogen receptor α; GSIS: glucose-stimulated insulin secretion; INS-1E: rat insulinoma β-cell line (clone 1E); IP3R2: inositol 1,4,5-trisphosphate receptor type 2; KO: knockout; MAM: mitochondria-associated membrane; Mfn2: mitofusin 2; MIN6 (Min6-B1): mouse insulinoma 6 pancreatic β-cell line (B1 subclone); MOTS-c: mitochondrial open reading frame of the 12S rRNA type-c; mRNA: messenger ribonucleic acid; nPOD: Network for Pancreatic Organ Donors with Diabetes; PERK: protein kinase R (PKR)-like endoplasmic reticulum kinase; PLA: proximity ligation assay; T1D: type 1 diabetes; T2D: type 2 diabetes; UPR: unfolded protein response; VDAC1: voltage-dependent anion channel 1).

**Table 5:** Therapeutic mechanisms targeting the ER–mitochondria axis in β-cells: potential targets versus strategies with therapeutic evidence. Evidence type indicates whether the supporting data derive from cellular studies, animal models, or patients.

Approach/Agent	Proposed Action on the ER–Mitochondria Axis	Evidence Type	Classification	Reference(s)
GLP-1 receptor agonists	Enhance ER–mitochondria coupling via PKA-dependent Mfn2 upregulation, increasing mitochondrial Ca^2+^ uptake, ATP and oxygen consumption	Patient (approved anti-diabetic use); cellular/animal for the MAM mechanism (non-β-cell: vascular smooth muscle)	Approved therapy; the ER–mitochondria mechanism itself remains preclinical and largely non-β-cell	[191,192,193,194,195,196]
Estrogen receptor α (ERα)	Preserves mitochondrial fission/fusion and ER morphology, limits ROS, supports MAM organization	Animal (ERα-knockout mouse islets) and cellular (ERα-silenced MIN6 cells)	Potential therapeutic target (preclinical, β-cell models)	[51]
PERK (partial inhibition)	Calibrates UPR output; improves cytosolic Ca^2+^ transit and glucose-stimulated insulin secretion	Animal (mouse islets) and ex vivo human islets	Potential therapeutic target (preclinical + ex vivo human)	[97]
PDZD8 (tether reduction)	Lowers excessive ER–mitochondria tethering, reducing organelle stress	Cellular/animal (experimental diabetic β-cell conditions)	Potential therapeutic target (preclinical)	[60]
MOTS-c (mitochondrial-derived peptide)	Restores MAM-associated Ca^2+^/redox pathways; reduces islet senescence	Animal (aged/diabetic mice) plus patient biomarker (reduced circulating MOTS-c in T2D)	Emerging strategy/target (preclinical animal + human correlative)	[190]

Ca^2+^: calcium ion; ER: endoplasmic reticulum; ERα: estrogen receptor α; GLP-1: glucagon-like peptide-1; MAM: mitochondria-associated ER membrane; Mfn2: mitofusin 2; MIN6: mouse insulinoma 6 pancreatic β-cell line; MOTS-c: mitochondrial open reading frame of the 12S rRNA type-c; PDZD8: PDZ domain-containing protein 8; PERK: protein kinase R-like ER kinase; PKA: protein kinase A; ROS: reactive oxygen species; T2D: type 2 diabetes; UPR: unfolded protein response.

## Data Availability

No new data was generated in the writing of this review article.
